# Isolation and characterization of *Lactobacillus*-derived membrane vesicles

**DOI:** 10.1038/s41598-018-37120-6

**Published:** 2019-01-29

**Authors:** Scott N. Dean, Dagmar H. Leary, Claretta J. Sullivan, Eunkeu Oh, Scott A. Walper

**Affiliations:** 1 National Research Council Associate, Washington, DC USA; 2 Center for Bio/Molecular Science & Engineering (Code 6900), US Naval Research Laboratory, Washington, DC USA; 3 Air Force Research Laboratory, Materials and Manufacturing Directorate, Wright-Patterson Air Force Base, Ohio, USA; 4 Sotera Defense Solutions, Inc, Columbia, MD USA

## Abstract

Bacterial membrane vesicles have been implicated in a broad range of functions in microbial communities from pathogenesis to gene transfer. Though first thought to be a phenomenon associated with Gram-negative bacteria, vesicle production in *Staphylococcus aureus*, *Lactobacillus plantarum*, and other Gram-positives has recently been described. Given that many *Lactobacillus* species are Generally Regarded as Safe and often employed as probiotics, the engineering of *Lactobacillus* membrane vesicles presents a new avenue for the development of therapeutics and vaccines. Here we characterize and compare the membrane vesicles (MVs) from three different *Lactobacillus* species (*L*. *acidophilus* ATCC 53544, *L*. *casei* ATCC 393, and *L*. *reuteri* ATCC 23272), with the aim of developing future strategies for vesicle engineering. We characterize the vesicles from each *Lactobacillus* species comparing the physiochemical properties and protein composition of each. More than 80 protein components from *Lactobacillus*-derived MVs were identified, including some that were enriched in the vesicles themselves suggesting vesicles as a vehicle for antimicrobial delivery. Additionally, for each species vesicular proteins were categorized based on biological pathway and examined for subcellular localization signals in an effort to identify possible sorting mechanisms for MV proteins.

## Introduction

Bacterial outer membrane vesicles (OMVs), proteoliposomes shed from the outermost membrane of Gram-negative bacteria, were observed in some of the earliest electron microscopy images of Gram-negative bacteria but were not believed to have a functional role at that time^1,2^. Years later interest in these structures sparked an ever-growing field of science that has been enabled, in part, by the development of technologies that allow interrogation of nanoscale structures in solution^3^. Continued efforts in vesicle research have indicated that these biological nanoparticles have a critical role in cellular function and community interaction. These observations are not limited to any specific bacterial species rather, membrane vesicles from Gram-positive bacteria (MVs), exosomes from eukaryotic cells, and OMVs have been shown to have an impressive range of extracellular function^4^. The OMVs of Gram-negative bacteria, which have been the most extensively studied vesicles thus far, have been shown to carry a wide range of cargo including virulence factors, nucleic acids, quorum sensing signals, toxins, immunomodulatory factors, adhesins, chelating molecules, and nutrient scavenging factors^5–7^. As an extension of the cargo they carry, OMVs have been associated with cytotoxicity, the invasion of host cells, membrane fusion, the production of biofilms, and the delivery of varied complex and simple biomolecules^8,9^. This diversity of composition and function suggests that OMVs and other vesicles have the potential to play a major role in the microbiomes in which they are produced^5,10^.

Gram-positive and Gram-negative bacteria differ significantly in the organization of their membrane and peptidoglycan layers which has contributed to the theories pertaining to membrane vesicle production. In Gram-negative bacteria, OMVs form on the outer membrane, drawing in components of the periplasm either passively or through some yet to be explained mechanism before being released into the surrounding environment^11^. Historically, Gram-positive bacteria were believed to not produce membrane vesicles due to their thick cell wall, seen as a potentially insurmountable barrier for vesicle release. Early skepticism was dispelled, however, when Lee *et al*. purified and characterized MVs isolated from *Staphylococcus aureus*^12^. Similar to the OMVs of Gram-negative bacteria, the MVs of *S*. *aureus* ranged in size from 20–100 nm and were shown to contain many proteins critical to the survival and pathogenesis of the bacterium. Subsequent to these studies, MVs have been isolated from several other Gram-positive bacteria, including *Streptomyces lividans*^13^, *Listeria monocytogenes*^14^, *Bacillus subtilis*^15^, *Lactobacillus plantarum*^16^ and *Lactobacillus reuteri* DSM 17938^17^.

Most lactobacilli are considered non-pathogenic and Generally Regarded as Safe (GRAS). Combined with their ability to grow aerobically or anaerobically, and lactic acid fermenting form of metabolism, these bacteria and others of their genus have been exploited for fermentation and food production for decades^18^. Additionally, the beneficial and probiotic effects of lactobacilli have been under investigation in both laboratory and clinical studies, with some studies finding a significant impact on human health^19^. Given their intriguing properties, MVs, and especially those produced by probiotic bacteria, may be an interesting avenue for various applications, from vaccines to therapeutic delivery^10,20^. In this study, we provide a detailed initial characterization of MVs from *L*. *acidophilus* ATCC 53544, *L*. *casei* ATCC 393, and *L*. *reuteri* ATCC 23272, with specific attention to the physicochemical and proteomic characterization with a goal of identifying characteristics or components that may subsequently prove useful for the engineering of the MVs themselves. This study will serve as a foundation for future efforts to understand the behavior of probiotic organisms, the role of bacterial MVs, and the potential for bacterially-derived, engineered therapeutics.

## Results

### Physicochemical characterization of MVs produced by *Lactobacillus* species

We first investigated whether *Lactobacillus* species under investigation in this study shed MVs. Recent reports have shown that both *L*. *plantarum* WCFS1^16^ and *L*. *reuteri* DSM 17938^17^ produce membrane vesicles, but it is currently unknown whether this is a widely conserved phenomenon within the genus. Here we examined each of the three *Lactobacillus* species at a late log stage of growth (60 hours) as determined by growth curve for each samples (data not shown). While earlier time points that corresponded to early and mid-log (20 and 40 hour, respectively) were also examined, these samples did not yield sufficient MVs for consistent proteomic analysis. Therefore, all analysis reported herein pertains to the 60 hour time point only.

**Figure 1:**
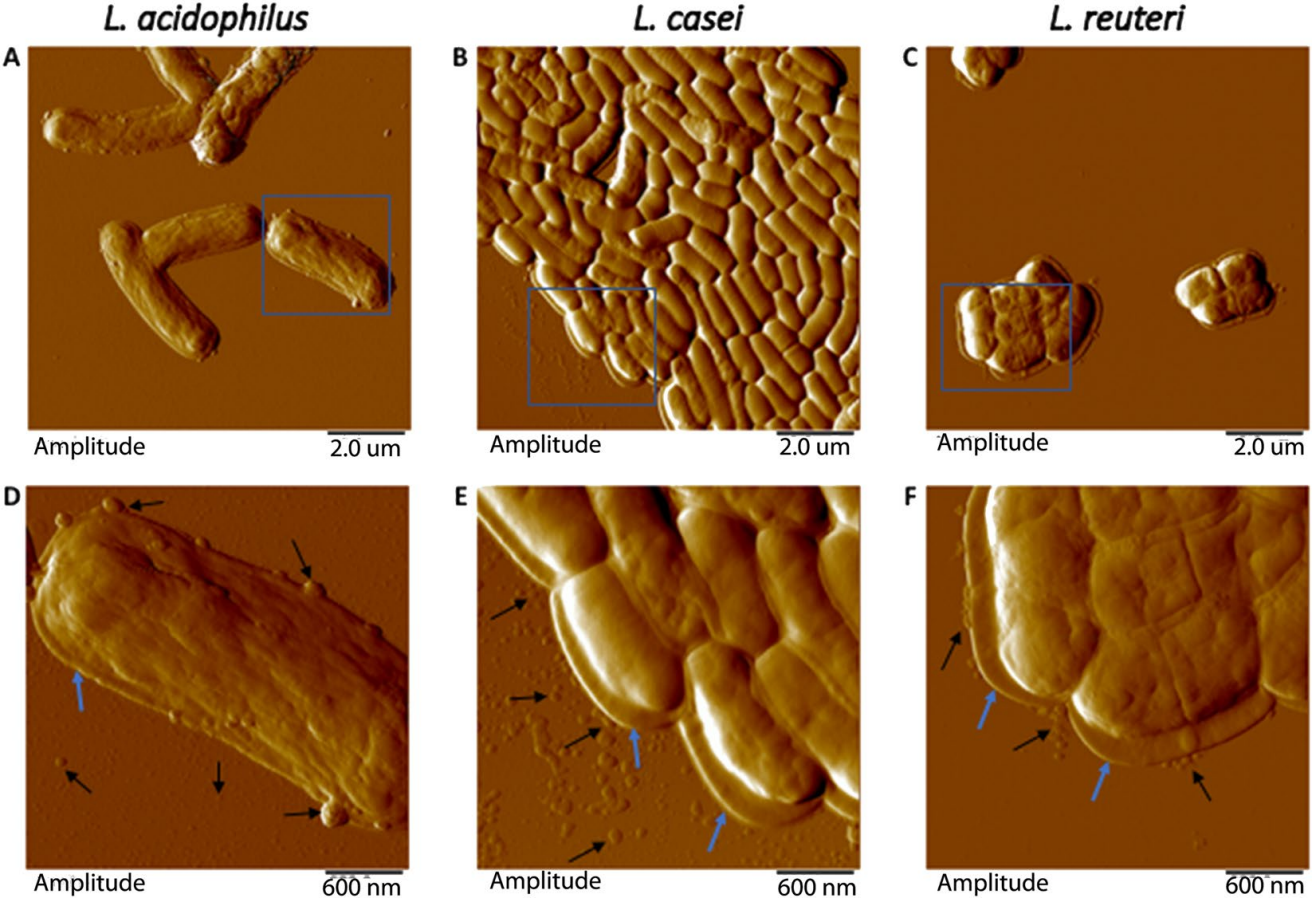
Representative atomic force microscopy (AFM) amplitude images of *Lactobacilli* and their associated membrane vesicles. (**A**–**C**) 10 micron scans of (**A**) *L*. *acidophilus*, (**B**) *L*. *casei*, (**C**) *L*. *reuteri*. In addition to having fewer cell-to-cell associations, the morphology of *L*. *acidophilus* varies considerably with respect to cell length and surface roughness when compared to *L*. *casei* and *L*. *reuteri*. (**D**–**F**) Six micron scans of the respective insets in (**A**–**C)** showing that vesicles are either associated with or proximal to the cells (black arrows). Given that peptidoglycan is cylindrical and the rigid part of the cell, it contributes to cell height in AFM images. These *Lactobacillus* species routinely have an additional material surrounding them (blue arrows). Although likely obscured by neighboring cells, the material is clearly visible in isolated or perimeter cells.

Images of the parental bacteria and both the nascent and released MVs were captured using atomic force microscopy (AFM). This method of imaging was chosen to minimize changes in cellular structures and the vesicles themselves. The facile sample preparation for AFM, which does not require vacuum conditions or gold coating as necessary for scanning electron microscopy (SEM), was determined to be the best method of imaging since the MVs would lack the peptidoglycan layer that lends rigidity to the parental cells. In all instances, spherical particles proximal to bacteria were observed (Figs 1, S1 and S2). The vesicles produced appear to be closed membrane structures, ranging in size from 10–300 nm (Fig. S3). These measurements were consistent for each of the three species and comparable to the MV/OMVs from other characterized Gram-negative and Gram-positive bacteria described in the literature^12^.

**Figure 2:**
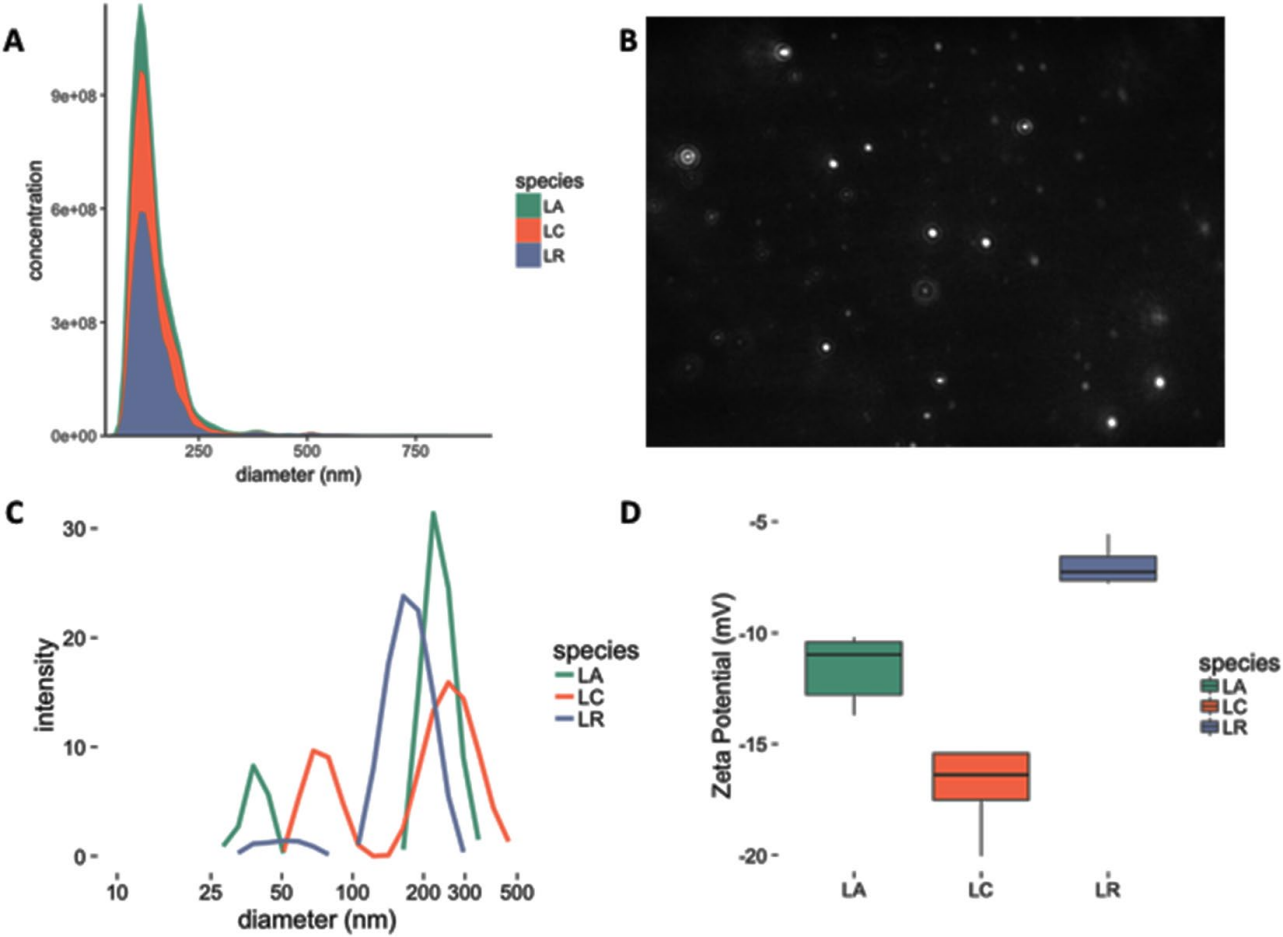
(**A**) MV size distribution was assessed on a NanoSight LM10 particle tracking system. (**B**) Representative frame from one of the *L*. *acidophilus* NanoSight videos is shown. Purified MVs were diluted in 1:100 or 1:1000 in PBS. (**C**,**D**) DLS was used to assess the (**C**) MV size distribution and (**D**) Zeta-potential of the *Lactobacillus* MVs in solution. Purified MVs were diluted in 0.1 × PBS. Measurements were performed in triplicate.

To further characterize the vesicles, we next purified MVs from each *Lactobacillus* species according to methods for purification of OMVs from Gram-negative species^21,22^. Purified samples were analyzed using NanoSight particle tracking instrumentation and software to obtain relative size distribution and approximate MV concentration. Using this protocol, MV concentrations typically ranged from 3 × 10^9^ to 1 × 10^10^ MVs/mL, approximately 10 to 100-fold less than what is typically seen in *E*. *coli*^22^. Analysis showed the mean sizes were 142 ± 64 nm, 143 ± 52 nm, and 143 ± 55 nm (all *n* ≥ 10000) with a right-skewed unimodal distribution, for *L*. *acidophilus* ATCC 53544, *L*. *casei* ATCC 393, and *L*. *reuteri* ATCC 23272, respectively (Fig. 2A). Analysis of AFM captures indicated a significant population of particles ranging from 25–50 nm in diameter, dimensions considered too small for accurate quantitation using NanoSight instrumentation. Secondary sample analysis using dynamic light scattering (DLS) was performed to better capture the population of smaller proteoliposomes. Interestingly, DLS analysis of the samples showed a bimodal distribution for each species, with a population of MVs sized between 20 and 100 nm, distinct from the MVs sized between 100 and 500 nm (Fig. 2C). The results are largely in agreement with the sizing analysis performed by Grande *et al*. who found *L*. *reuteri* DSM 17938 MVs were multimodal by DLS but unimodally distributed using NanoSight^17^. When paired with the lower enumeration of particles than what is normally seen in *E*. *coli*, the difference in sizing result obtained by the two methods may be caused by the NanoSight’s high minimum particle size of approximately 50 nm. However, we cannot exclude the possibility of contaminating cellular debris. Additional data from the NanoSight analysis including volume and surface area for each species is included in supplemental materials (Fig. S4), which further supports the conclusion that MVs produced by *Lactobacillus* species are polydisperse, in contrast to what the concentration distribution output by NanoSight suggests. Finally, Zeta-potential measurements and electrophoretic mobility data were acquired to further characterize the physiochemical properties of the *Lactobacillus* MVs. The MVs for all three species showed a net negative charge and corresponding electrophoretic mobility (Figs 2 and S5). These values are consistent with those previously reported for Gram-negative OMVs and Gram-positive MVs, including *Bacillus subtilis* and *L*. *reuteri*^15,17^, and consistent with the net negative charge of the bacterial cell surface^23^. However, between species significant differences in Zeta-potential and electrophoretic mobility were seen (*p* < 0.01), with no significant difference in conductivity (ranging between 2.3 and 2.8 mS/cm) (Fig. S5).

### Proteomic analysis of *Lactobacillus*-derived MVs

**Figure 3:**
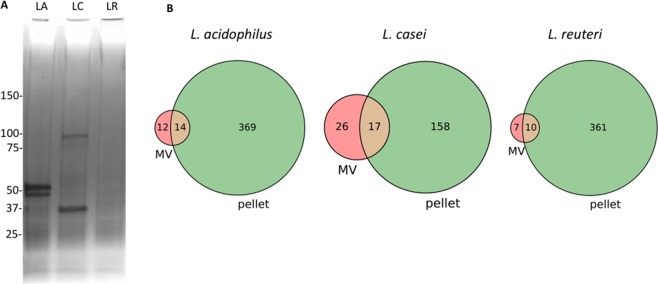
Protein composition of *Lactobacillus* MVs. (**A**) Gel-Code Blue-stained SDS-PAGE of purified *L*. *acidophilus* (LA), *L*. *casei* (LC), *and L*. *reuteri* (LR). MVs with equal number of MVs loaded. For the sake of clarity the *Escherichia coli* OMVs that were run in parallel were removed from this image. A complete gel image can be found in the Supplemental Material. (**B**) Venn diagrams of the identified proteins that are unique or in common between the MVs and pellets of each *Lactobacillus* species.

In many instances, the composition and payload of membrane vesicles provides some insight as to their role or function. While limited to laboratory conditions, the protein composition of both intact bacterium and the purified MVs for each of the three bacterial species was determined using a combination of qualitative biochemical analysis and more definitively via mass spectrometry. Purified MVs from all three bacterial species were first examined via SDS-PAGE. Analysis showed species-specific banding, with *L*. *acidophilus* ATCC 53544 and *L*. *casei* ATCC 393 both displaying two prominent though dissimilar bands. In contrast, no prominent bands were observed within the *L*. *reuteri* ATCC 23272 lane (Figs 3A and S6). Efforts were made to ensure uniform loading of samples based on the measured MV concentration (MV/mL). Elevated concentrations of some membrane proteins is not uncommon and has been observed with other bacterial species, less common is the lack of definitive bands as was seen with *L*. *reuteri* ATCC 23272.

Protein composition of *Lactobacillus*-derived MVs and the corresponding cell pellets was more definitively assayed via shotgun proteomics. For these studies, all samples were prepared from three biological replicates. Proteins identified by at least 2 peptides were considered; see Materials and Methods for more details. A total of 395 proteins from *L*. *acidophilus* ATCC 53544 (of which 26 were vesicular proteins), 201 proteins for *L*. *casei* ATCC 393 (43 vesicular proteins), and 378 proteins for *L*. *reuteri* ATCC 23272 (17 vesicular proteins) were identified. The low number of vesicular proteins identified for the *L*. *reuteri* ATCC 23272 sample is consistent with the absence of bands visualized via SDS-PAGE. Venn diagrams in Fig. 3B show the overlap between proteins identified in the pellet and the MVs for the three species. Principal component analysis performed on normalized weighted spectral counts show clear separation between MV and pellet samples with low variance between biological replicates within each group (Fig. S8). Protein identification information for all proteins is presented in Table S1 of the Supporting Information.

Quantitative analysis was performed on normalized weighted spectra to compare protein composition of the analyzed samples. Proteins were sorted by fold difference in weighted spectra (MV/pellet) for each of the sample examined, Table 1. Among the *L*. *acidophilus* ATCC 53544 MV proteins with the highest spectral count were Mucus binding protein (Mub), putative bacteriocin LBA1805, surface protein FmtB, inducing peptide IP1800, and surface layer protein SlpX. For *L*. *casei* ATCC 393, a putative family 14 glucoamylase, cell-wall associated hydrolase, and two proteins annotated as lysozyme were the proteins with the highest counts in the MVs. For *L*. *reuteri* ATCC 23272, which contained notably fewer proteins, NAD kinase NadK and metabolic proteins were higher in purified MVs. One commonality between all three *Lactobacillus* species was the identification of elongation factor Tu (EF-Tu) as the protein with the highest spectral count in the pellet, while none was present in their corresponding MVs (Tables S1–3). For comparison, Lee *et al*. found that EF-Tu was the most abundant vesicular protein in MVs purified from *S*. *aureus*^12^. The notable lack of EF-Tu in *Lactobacillus* MVs also contrasts with the presence of the protein in OMVs of several pathogenic Gram-negatives, including *E*. *coli* isolates from hospital patients^24^, *Burkholderia pseudomallei*^25^, and *Acinetobacter baumannii*^26^, where it has been implicated in the pathogenesis of bacterial infection through its ability to adhere with host cells and its immunomodulatory effects. Interestingly, EF-Tu in some *Lactobacillus* species has been shown to be cell membrane associated where it plays a role in the attachment of the bacterium to the human intestinal epithelium^27^. The lack of EF-Tu in the MVs suggests that EF-Tu in these species may not be membrane associated and could contribute to the allochthonous nature of each of these bacterial species. Finally, in a recent study by Li *et al*., EF-Tu was also found to be absent in MVs purified from *L*. *plantarum* WCFS1; despite this, the group did observe upregulated expression of host defense genes which provide protective benefits to the host when exposed to *L*. *plantarum* MVs^16^, suggesting that *Lactobacillus* MVs may interact with the host differently, relative to previously studied MVs and OMVs produced by pathogenic bacteria.

**Table 1:** List of top proteins identified in *Lactobacillus* MVs, sorted by fold difference in weighted spectral counts (MV/pellet).

Description	UniProt ID	Gene	MW	Fold change	MV average	Pellet average	p-value
*L. acidophilus*							
Mucus binding protein Mub	Q5FJA7_LACAC	LBA1392	466 kDa	608.89	182.67	0.30	0.08
Putative uncharacterized protein	Q5FI65_LACAC	LBA1805	6 kDa	513.33	154.00	0.30	0.01
Surface protein fmtB	Q5FIP8_LACAC	LBA1611	268 kDa	51.44	15.43	0.30	0.12
Cell division protein DivIB	Q5FKV1_LACAC	LBA0810	32 kDa	40.67	12.20	0.30	0.37
Putative uncharacterized protein	Q5FIG. 5_LACAC	LBA1697	37 kDa	40.67	12.20	0.30	0.37
Glutamine ABC transporter permease protein glnP	Q5FMN9_LACAC	LBA0134	54 kDa	40.67	12.20	0.30	0.37
ABC transporter ATP-binding and membrane spanning protein	Q5FHZ8_LACAC	LBA1876	59 kDa	40.67	12.20	0.30	0.37
Signal peptide IP_1800	Q5FI70_LACAC	LBA1800	5 kDa	31.47	73.43	2.33	0.13
Surface layer protein X SlpX	Q5FLN0_LACAC	LBA0512	54 kDa	28.92	115.67	4.00	0.01
Maltose ABC transporter permease protein	Q5FI08_LACAC	LBA1866	44 kDa	27.33	8.20	0.30	0.37
*L. casei*							
putative family 15 glucoamylase	S6CK93_LACCA		101 kDa	431.11	129.33	0.30	0.04
putative major head protein	S6C5N2_LACCA		42 kDa	420.00	126.00	0.30	0.01
putative cell wall-associated hydrolase	Q03CD1_LACP3		48 kDa	176.51	741.33	4.20	0.00
conserved hypothetical protein	Q03BH5_LACP3		32 kDa	117.00	35.10	0.30	0.22
ATP synthase gamma chain	ATPG_LACP3	atpG	34 kDa	93.33	28.00	0.30	0.01
Translation initiation factor IF-2 infB	IF2_LACP3	infB	103 kDa	64.44	19.33	0.30	0.02
lysozyme M1 (1,4-beta-N-acetylmuramidase)	Q03CH3_LACP3		75 kDa	60.67	18.20	0.30	0.37
capsid protein	S6C5N2_LACCA		42 kDa	59.56	17.87	0.30	0.37
N-acetylmuramoyl-L-alanine amidase	Q038R1_LACP3		47 kDa	58.11	17.43	0.30	0.17
putative lysozyme	Q03CH3_LACP3		74 kDa	45.15	155.00	3.43	0.02
*L. reuteri*							
NAD kinase nadK	NADK_LACRD	Lreu_0573	31 kDa	76.22	22.87	0.30	0.37
Uncharacterized protein	A5VIP4_LACRD	Lreu_0450	50 kDa	67.33	20.20	0.30	0.37
Cobalamin biosynthesis protein CobD	A5VM86_LACRD	Lreu_1721	36 kDa	38.44	11.53	0.30	0.37
PTS system IIA component, Glc	A5VKG9_LACRD	Lreu_1086	70 kDa	35.89	10.77	0.30	0.12
RNA binding S1 domain protein	A5VI82_LACRD	Lreu_0286	82 kDa	29.56	8.87	0.30	0.37
Phage tape measure protein	A5VKJ0_LACRD	Lreu_1107	143 kDa	19.56	5.87	0.30	0.37
ABC transporter related	A5VLR9_LACRD	Lreu_1548	35 kDa	19.56	5.87	0.30	0.37
Mannosyl-glycoprotein endo-beta-N-acetylglucosamidase	A5VML7_LACRD	Lreu_1853	60 kDa	11.71	82.00	7.00	0.05
Carbamate kinase	A5VIM1_LACRD	Lreu_0426	33 kDa	5.75	226.00	39.33	0.02
Signal recognition particle protein ffh	A5VKN8_LACRD	Lreu_1155	54 kDa	3.32	5.87	1.77	0.51

### Subcellular localization of membrane vesicle proteins

Most secreted, surface, and periplasmic proteins are known to have a signal peptide sequence in their N-termini marking them for localization to the appropriate cellular location. For the three species in the study, the signal peptide predictor SignalP 4.1 was used to categorize the proteins identified in the MVs and cell pellet based on the presence of a signal peptide. In each of the species examined a higher proportion of vesicular proteins contained signal peptides than in the cell pellet. In *L*. *acidophilus* ATCC 53544, 9 (35% of the total) vesicle proteins contained a signal peptide; 13 (30%) and 3 (18%) for *L*. *casei* ATCC 393 and *L*. *reuteri* ATCC 23272, respectively (Table 2). Conversely, the proteins identified in the cell pellet largely did not contain signal peptides, where between 93 and 98% of the proteins in those samples were predicted to have no signal peptide.

**Table 2:** Proportion localization of proteins from each sample in the study using SignalP^46^ and LAB-Secretome Database^28^.

	LA MVs	LA pellet	LC MVs	LC pellet	LR MVs	LR pellet
SignalP						
signal peptide	0.35	0.07	0.30	0.06	0.18	0.02
no signal peptide	0.65	0.93	0.70	0.94	0.82	0.98
LAB-SecretomeDB						
Intracellular	0.62	0.91	0.65	0.94	0.82	0.96
Lipid anchored	0.12	0.02	0.12	0.03	0.00	0.01
N-terminally anchored (No CS)	0.04	0.02	0.05	0.00	0.06	0.02
N-terminally anchored (with CS)	0.04	0.01	0.02	0.01	0.12	0.01
Secretory(released) (with CS)	0.12	0.03	0.14	0.02	0.00	0.01
LPxTG Cell-wall anchored	0.08	0.00	0.02	0.00	0.00	0.00

Cleavage site = CS.

For more detailed localization analysis, we annotated a total of 974 proteins based on LAB-SecretomeDB annotation, a database that provides subcellular localization for 26 sequenced lactic acid bacteria genomes^28^. Proteins were broadly grouped into intracellular and membrane/cell-wall associated. As the focus of these efforts is to identify and characterize the proteins of the MVs, the membrane/cell wall-associated was further sub-divided into categories that provide additional insight as to the mechanism of protein anchoring to the exterior of the bacterium. The percentage of proteins in each category, both for MV and cell pellet samples is reported in Table 2. In all three *Lactobacillus* species examined, proteins of the cell pellet were largely categorized as intracellular, as between 91 and 96% were categorized as intracellular. Examined broadly, the categorization of the proteins largely matches the categorization by SignalP, where the percentage labeled “intracellular” and “no signal peptide” are similar.

While to date there is not extensive characterization of the MVs of Gram-positive bacteria, it was expected that a significant proportion of the protein composition would be cytoplasmic proteins that are passively packaged into the MV during formation. Differences in cell wall structure between Gram-negatives and Gram-positives would lead one to expect sequestration of distinct proximal cellular components. Specifically, OMVs would more likely contain periplasmic constituents whereas MV constituents would likely originate from the cytoplasm. Indeed previous studies of OMVs and supportive^29,30^. In our analysis of *Lactobacillus* MVs, between 62 and 82% of identified proteins are categorized as intracellular, non-membrane associated proteins, consistent with the theory of passive periplasmic loading of MVs. Additionally, for each of the *Lactobacillus* species, the identified vesicular proteins showed a varied distribution into the secreted or membrane and cell wall-associated categories. Therefore, a significant proportion of the MVs protein composition was categorized as secretory or released proteins. In fact, proteins within these categories were several-fold higher than what was found in the pellet, suggesting that secreted proteins can either be contained within or associated with the membrane of the MVs. These data are consistent with other proteomic analyses of Gram-positive MVs that saw an increased proportion of proteins containing Sec and Tat signal peptides^12,13^ suggesting that the location of MV formation may correlate with higher levels of secretion machinery or proteins prior to release from the cell, analogous to the increased presence of periplasmic proteins reported in Gram-negative OMVs. Overall, however, despite the elevated protein levels for secreted, cell wall-associated, and membrane categorized proteins; these results suggest that at the time point and conditions used in this study, there does not appear to be a packaging mechanism by which proteins are specifically exported via or concentrated within *Lactobacillus* MVs.

### Functional classification of membrane vesicle proteins

Previous proteomic analyses have found that MVs can contain enzymes and complete, active metabolic pathways which may enable functions external to the cell^31^. In this study, the functions of >150 proteins identified from MV and pellet samples from each species were categorized according to KEGG pathways^32^. Proteins were selected following calculation of the fold difference in normalized weighted spectral count between MVs and pellet of the same species. Additionally, based on the analysis of the proteomic data, we added the category “Bacteriocin pathway” in order to categorize a subset of proteins thought to be associated with this microbial pathway. These proteins were then compared to previously reported bacteriocins and signaling peptides of *Lactobacillus*^33,34^.

**Figure 4:**
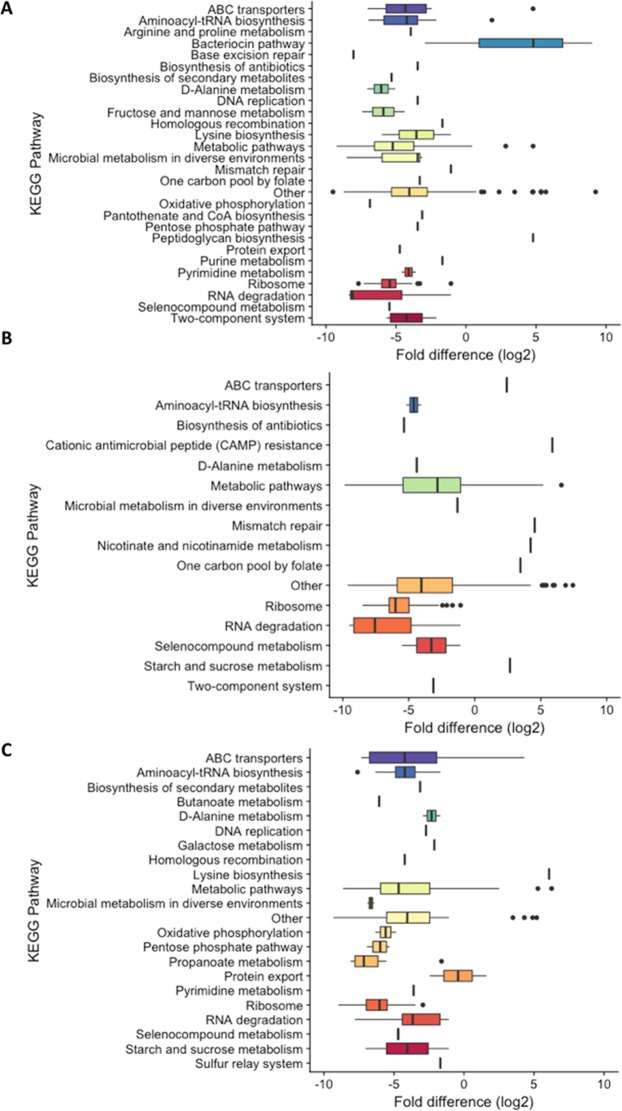
Functional (KEGG) categorization of normalized weighted spectral counts for (**A**) *L*. *acidophilus*, (**B**) *L*. *casei*, and (**C**) *L*. *reuteri*. All proteins were categorized into KEGG categories, and the fold-changes (count in MVs/count in pellet) from the proteomics analysis are plotted. Weighted spectral counts used are averages from three separate experiments. The full proteomics tables are provided in Tables S1–S3 in the supplemental material. CoA, coenzyme.

There was little consistency between the protein compositions of the MVs from each of the *Lactobacillus* species examined. In *L*. *acidophilus* ATCC 53544, components of the bacteriocin pathway, including putative bacteriocin LBA1805 and inducer peptide IP1800, were significantly enriched when compared to the protein composition of remaining cell pellet. On average, the bacteriocin and auto-inducer peptide were 182-fold higher in the MVs than the pellet (discussed in greater detail below). As expected, proteins from most other pathways were substantially less likely to be identified within the MVs. Those proteins and enzymes most closely associated with metabolic pathways were down 2-fold, RNA degrading proteins were down 5-fold, and ribosomal proteins were approximately 25-fold lower. Boxplots for fold difference of each KEGG pathway are shown in Figs 4 and S7.

Proteomic analysis of *L*. *casei* ATCC 393 and *L*. *reuteri* ATCC 23272 did not suggest an enrichment of any particular protein or components of a specific metabolic pathway. To the contrary, certain proteins were significantly less likely to be found in the MVs. Though further studies would be necessary, these findings suggest that most of the MV-associated proteins as passively packed during MV formation.

### Bacteriocin pathway proteins in MVs of *L*. *acidophilus*

An interesting group of proteins identified in the MVs of *L*. *acidophilus* ATCC 53544 are involved in bacteriocin production and signaling. Bacteriocins are small antimicrobial peptides produced widely in lactic acid bacteria^35^. Proteins identified with the highest probability and spectral count in *L*. *acidophilus* ATCC 53544 MVs were LBA1805 and LBA1800. In *L*. *acidophilus* NCFM, the ~10-kb region that makes up the *lab* operon contains several ORFs encoding components responsible for regulation, production, and export of bacteriocin and signaling peptides^33,36^, where LBA1805 encodes for a putative bacteriocin and LBA1800 encodes for the inducer peptide, IP1800^34,37^. IP1800 was very low and LBA1805 was not detected in the corresponding *L*. *acidophilus* ATCC 53544 bacterial pellet, resulting in a fold difference of 31 and 513, respectively (Table 1). Though our shotgun proteomics approach was sufficient for a broad survey of MV-associated proteins, further analysis of the data was required to determine of the presence of IP1800 in MV sample 2. We found that the 460.25 Da peptide had slightly different elution time in MV sample 2. This inhibited detection in some samples and excluded the peptide from MS/MS analysis (Fig. S9). This complication likely skewed the calculated value of a 31-fold increase in abundance of IP1800 in *L*. *acidophilus* ATCC 53544 MVs which may be higher than reported here. Interestingly, neither *L*. *casei* ATCC 393 nor *L*. *reuteri* ATCC 23272 MVs and pellets contained detectible bacteriocin or signaling peptides. Consistent with other findings, *L*. *casei* ATCC 393 may not produce bacteriocin, due to an interrupted ABC transporter^38^, however, since *L*. *reuteri* ATCC 23272 has been used as a positive control for bacteriocin production and is a clear producer of the bacteriocin reuterin^39^ its absence may indicate differing protein or peptide packaging between *Lactobacillus* species.

The identification of IP1800 and a putative bacteriocin in *L*. *acidophilus* ATCC 53544 may have interesting implications for signaling and community control in *Lactobacillus*. It has been reported that OMVs can facilitate the intercellular trafficking of diffusible quorum sensing signals through their packaging into vesicles many prokaryotes^40,41^, however, this has not been previously shown in Gram-positive bacteria. The dearth of literature on the trafficking of signals via MVs in bacteria may be due to the relatively recent acknowledgment of MV production in Gram-positives. For *L*. *acidophilus* ATCC 53544, this finding suggests that the diffusion of IP1800 and bacteriocin, known to influence the local microbial ecology^33,37^, may be facilitated by MVs. These findings may aid in the understanding of intra-/inter- cellular communication and material transfer in *Lactobacillus* as well as the role these signals and the vehicles that carry them play in larger microbial communities such as those of the gut.

## Discussion

This study provides a detailed characterization of the MVs from three species of *Lactobacilli; L*. *acidophilus*, *L*. *casei*, and *L*. *reuteri;* focusing primarily on physicochemical and proteomic characterization. The MVs of both commensal and pathogenic bacteria have been shown to the carry a wide range of cargo including virulence factors, quorum sensing signals, toxins, immunomodulatory factors, adhesins, and other factors, implicating MVs in the functions ranging from cytotoxicity to production of biofilms. Additionally, with growing evidence for interaction between gut microbial communities and the host, there is significant need to characterize not only the bacteria themselves but the MVs that are shed during the bacterial lifecycle.

Here we confirm the production of MVs by three *Lactobacillus* species (*L*. *acidophilus* ATCC 53544, *L*. *casei* ATCC 393, and *L*. *reuteri* ATCC 23272), characterizing some of their physiochemical properties including size distribution, charge, and protein composition. Under the growth conditions used here and at the late-log growth stage (60 hours) all three species produced MVs that ranged in size from 20–400 nm in size though the vast majority of these particles fell within a range of 20–50 nm and 100–150 nm following a bimodal distribution of particle size. This is consistent with the size distribution observed for other Gram-positive bacteria described in the literature. The relative number of MVs produced (concentration) and their charge was also consistent with those described in the literature for both Gram-negative and positive bacteria.

Though no specific MV packaging was identified, analysis of MV proteins and the presence and absence of signal peptides does suggest that these cellular signals do contribute to an improved probability of MV loading. While the majority of MV protein composition was intracellular proteins, each strain also showed a localization of proteins that possessed signal peptides that target proteins to the secretory pathway. This would need to be confirmed through molecular studies and recombinant expression of tractable proteins, however, these observations may serve as starting point for the engineering of *Lactobacillus* MVs.

Of significant interest to these and future studies was the elevated concentration of the antimicrobial bacteriocin in the MVs of *L*. *acidophilus* ATCC 53544. It has been postulated that MVs may serve as delivery vehicles for antimicrobial compounds^13^, affording protection to cargo proteins and possible mechanisms of targeted delivery. The significantly increased concentration of putative bacteriocins suggests a possible packaging mechanism is in play, though not identified here. Further characterization of the *lab* operon and the signal sequences preceding putative bacteriocin peptides may allow for the development of MV loading strategies that could include other antimicrobials or other proteins and peptides that could find a role in community regulation.

The three bacteria described here are all Generally Regarded as Safe and have been incorporated into a range of probiotic products with little understanding of their role in gut microbial communities. While this study was conducted with pure cultures under laboratory conditions, evidence for the species specific packaging of proteins and peptides to the MVs alludes to the bacterial utilization of these nanoscale proteoliposomes for community interaction. The findings presented here may stimulate future research into *Lactobacillus* MVs, MV-associated signaling and bacteriocin secretion in *L*. *acidophilus*, and serve as a foundation for future studies investigating the natural function of *Lactobacillus* MV or their potential applications as delivery vehicles.

## Materials and Methods

### Cell culture and purification of MVs

*Lactobacillus acidophilus* (ATCC 53544), *Lactobacillus casei* (ATCC 393), and *Lactobacillus reuteri* (ATCC 23272) were grown in de Man, Rogosa, Sharpe (MRS) broth under anaerobic conditions. Anaerobic conditions were maintained using AnaeroGen anaerobic atmosphere generation bags (Fluka, St. Louis, MO, USA) in AnaeroJar jars (Fisher, Hampton, NH, USA). Oxygen level (<1%) was monitored using anaerobic indicator strips (Fisher, Hampton, NH, USA). The procedure for purification of MVs from *Lactobacillus* culture supernatants was similar to methods previously described for OMV purification from *E*. *coli*^21^, with some modifications. Briefly, three 200 mL cultures were grown statically at 37 °C and collected at time points of 20, 40, and 60 hours for each *Lactobacillus* species. Cells were pelleted via centrifugation at 5000 x g and the supernatant decanted to a clean centrifuge bottle. Centrifugation was repeated two additional times after there was no visible pellet on the bottle walls, typically 4–6 cycles. The supernatant fraction was then passed through a 0.45 µm using a vacuum apparatus to ensure there were no residual cells in the supernatant fraction. A 36 mL volume of the filtrate was then ultracentrifuged at 129,000 × g for 1.5 h in a Sorvall WX Ultra 90 centrifuge using an AH-629 rotor (Thermo Scientific, Rockford, IL). The supernatant was decanted and the MV pellet, which is often invisible, was incubated overnight at 4 °C in PBS to resuspend the MVs. Subsequent to all purification steps, MVs were considered concentrated 36-fold compared to original culture medium.

### AFM

Bacteria used for atomic force microscopy (AFM) were grown to 60 h and fixed for 10 min with 0.25% glutaraldehyde. The samples were then pelleted and washed four times with water. To prepare samples for imaging, 1 µl of the glutaraldehyde-fixed suspension was diluted with 4 µl of water, applied to freshly cleaved mica, and allowed to dry. AFM was performed using a Bruker Dimension 3100 in tapping mode under ambient conditions. For imaging, silicon cantilevers (Nanosensors PPP-NCHR) having nominal spring constants and resonance frequencies of ~42 N/m and 330 kHz, respectively were used. Scan rates ranged from 0.5 Hz to 1.0 Hz with 512 data points per line. Image data scales were adjusted using Bruker’s Nanoscope analysis software.

### NanoSight

Vesicle count as well as size, volume, and surface area distributions were obtained on a NanoSight LM10 system (Malvern Instruments Ltd, Worcestershire, UK) using NTA 2.3 Nanoparticle Tracking and Analysis software. Samples were diluted 1:100 or 1:1000 in pH 7.4 PBS with camera shutter and gain optimized for data collection. Videos (90 s) were taken and frame sequences were analyzed under auto particle detection and tracking parameters: detection threshold, pixel blur, minimum track length, and minimum expected particle size. All samples were run at RT and allowed to equilibrate prior to analysis.

### Dynamic Light Scattering and Zeta-Potential

Dynamic light scattering (DLS) measurements were carried out using ZetaSizer NanoSeries equipped with a HeNe laser source (λ = 633 nm) (Malvern Instruments Ltd, Worcestershire, UK) and analyzed using Dispersion Technology Software (DTS, Malvern Instruments Ltd, Worcestershire, UK). MVs were loaded into disposable cells, and data were collected at 25 °C. All the samples were prepared in 0.1 x PBS buffer pH 7.4. For each sample, the autocorrelation function was the average of five runs of 10 seconds each and then repeated about three to six times. CONTIN analysis was then used to number *versus* hydrodynamic size profiles for the dispersions studied.

For Zeta-Potential (ζ-potential) measurement, Laser Doppler Velocimetry (LDV) measurements were performed using a ZetaSizer NanoSeries equipped with a HeNe laser source (λ = 633 nm) (Malvern Instruments Ltd, Worcestershire, UK) and an avalanche photodiode for detection, controlled with DTS software. MVs were loaded into disposable cells, and data were collected at 25 °C. Three runs of the measurements were performed for each sample to achieve the zeta potential. All the samples were prepared in 0.1 × PBS buffer pH 7.4. For both DLS and zeta-potential, MVs were used at a 10-fold dilution from stock concentration.

### SDS-PAGE

Equal numbers of purified MVs from each *Lactobacillus* species were analyzed with denaturing, polyacrylamide gel electrophoresis using a linear concentration gradient of polymer (4–15% Tris-glycine gel). Samples were denatured through boiling in the presence of a reducing agent (2-mercaptoethanol) prior to gel loading. The gel was subsequently stained with GelCode Blue Stain Reagent (Pierce, Rockford, IL).

### Proteomics analysis

Triplicate biological samples of MVs and bacterial pellets from *L*. *acidophilus*, *L*. *casei*, and *L*. *reuteri* were harvested at 60 h. Pellets were lysed using OneShot (Constant Systems Ltd., Daventry, UK) at 40 kpsi pressure in 10% n-propanol in 50 mM ammonium bicarbonate (ABC) in a 10 mL suspension. The instrument was then washed with 10 mL ABC, the lysate and wash were combined, and then evaporated via speed-vac. Samples were normalized by total protein content to 100 µg prior digestion using the Pierce BCA Protein Assay Kit (Thermo Scientific, Rockford, IL). All samples were digested in solution with sequencing-grade modified trypsin (Promega, Madison, WI) at a 1:30 w/w enzyme to substrate ratio in a barocycler (Pressure Biosciences Inc., Easton, MA) for 90 min (90 cycles: 50 s on at 20 kpsi, 10 s off). Digested samples (150 µL) were evaporated via speed-vac. MVs were solubilized in 10% n-propanol, digested in solution and dried as described above for pellets. All dried samples were stored at −20 °C until they were analyzed by LC-MS/MS. Immediately prior to analysis, samples were solubilized in solvent A (0.1% formic acid (FA) in HPLC grade water) and 10 µL of sample (~50 µg of total protein) was injected into the LC-MS/MS system (Tempo-MDLC coupled to a TripleTOF 5600 mass spectrometer - Sciex, Foster City, CA). Peptides were loaded for 15 min in 5% solvent B ((0.1% FA in acetonitrile) and 95% solvent A, separated on two eksigent C18 Chrom XP columns (150 × 0.3 mm, 120A) connected in a row using a linear gradient of increasing mobile phase B in the rate of 0.52% per minute. The 180 min LC method also included 10 min column wash at 80% B and re-equilibration of the columns with the starting condition at 5% solvent B.

Protein identifications were accepted if they could be established at greater than 90.0% probability and contained at least 2 identified peptides. Protein probabilities were assigned by the Protein Prophet algorithm^42^. Proteins that contained similar peptides and could not be differentiated based on MS/MS analysis alone were grouped to satisfy the principles of parsimony. Quantitative analysis was done in Scaffold using weighted spectra as an input. Only spectra satisfying the probability settings were considered for the analysis (lower scoring matches and probabilities < 5% were not included). More detailed methods for Proteomics analysis are provided in Supplemental Information.

In order to avoid divide-by-zero errors caused the absence of proteins in the MVs or pellet in fold difference calculations, we set missing values to 0.3, as previously described^43^. *t*-test, fold difference, and other calculations were performed on normalized weighted spectral counts using Scaffold^44^ and in-house R and Python scripts were used for principal component analysis, database mining, and annotation^45^.

## Supplementary information

Supplementary information

table s1

table s2

tablse s3

## Data Availability

The mass spectrometry proteomics data have been deposited to the ProteomeXchange Consortium via the PRIDE partner repository with the dataset identifier PXD011278 and 10.6019/PXD011278.
