# Supplementary material for: Isolation and characterization of Lactobacillus-derived membrane vesicles
Source: Sci Rep. 2019 Jan 29;9:877. doi: 10.1038/s41598-018-37120-6 (PMC6351534; doi:10.1038/s41598-018-37120-6)
Supplement: Supplementary file 1 — Supplementary information [file 41598_2018_37120_MOESM1_ESM.pdf]

Isolation and characterization of *Lactobacillus*-derived membrane vesicles

Scott N. Dean[1], Dagmar H. Leary[2], Claretta J. Sullivan[3], Eunkeu Oh [4], Scott A. Walper[2 *]

[1] National Research Council Associate, Washington, DC, USA

[2] Center for Bio/Molecular Science & Engineering (Code 6900), US Naval Research Laboratory, Washington DC, USA

[3] Air Force Research Laboratory, Materials and Manufacturing Directorate, Wright-Patterson Air Force Base, Ohio, USA

[4] Sotera Defense Solutions, Inc. Columbia, MD, USA

* Address correspondence to Scott A. Walper, scott.walper@nrl.navy.mil

Supplemental material

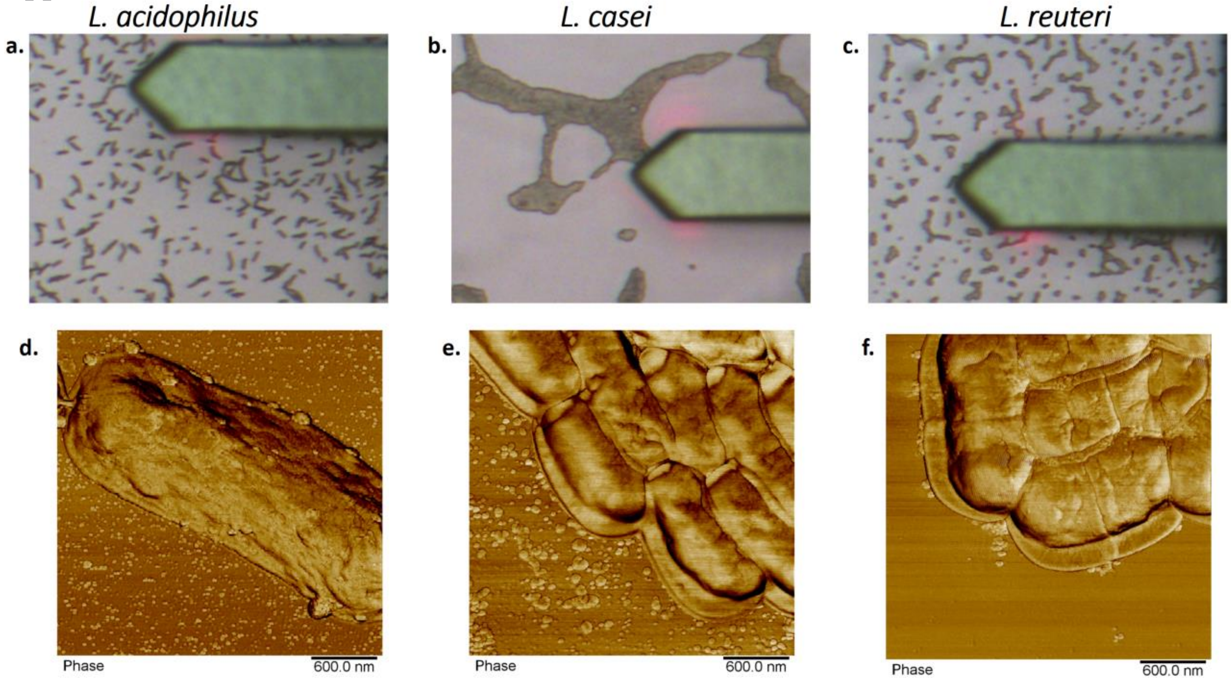

Fig. S1.
The AFM is equipped with an optical microscope to assist with locating the cantilever and selecting a region of interest. (a-c) Optical images of regions imaged in figure 1 show that *L. acidophilus* cells appear on the mica substrate more often as relatively large single cells whereas *L. casei and L. reuteri* were more likely to associate in 'pods' of cells. (d-e) Phase images that were acquired simultaneously with the amplitude images shown in figure 1, d-f.

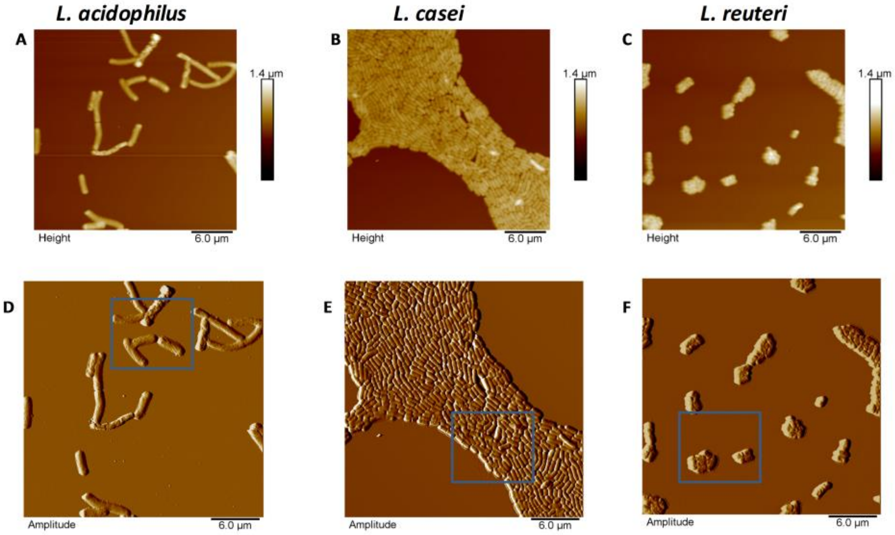

Fig. S2. Thirty micron height (A-C) and amplitude (D-F) images for a larger perspective of the regions shown in Fig. 1.

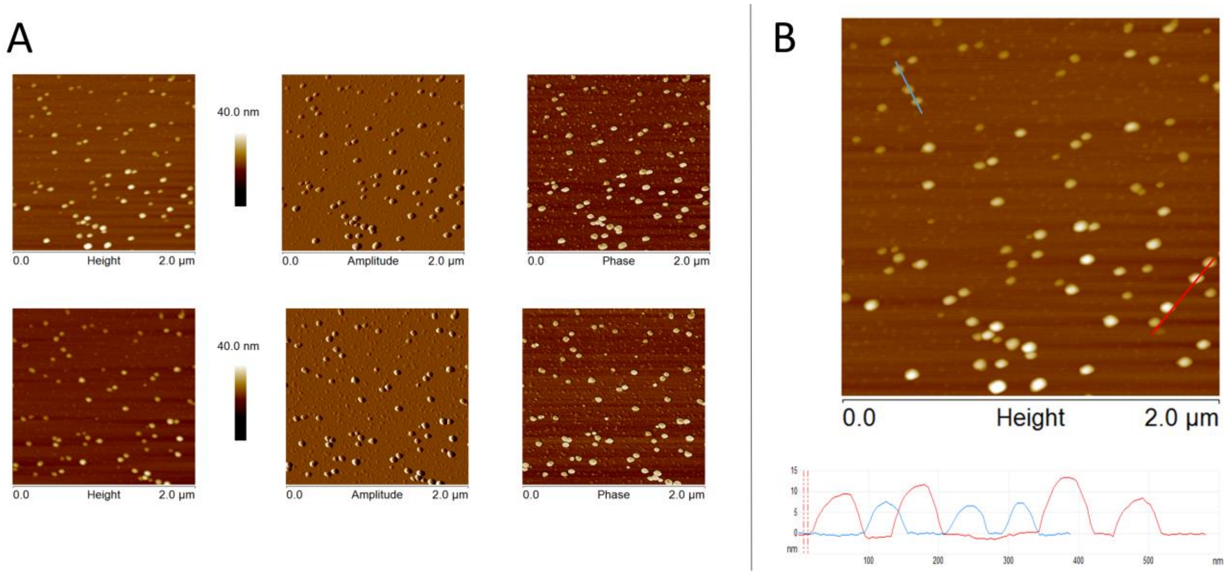

Fig. S3. Membrane vesicles from *L. casei* 60 h time point imaged using AFM (panel A). Isolated MVs were measured using instrument software and some a size distribution consistent with those results observed using the NanoSight particle tracking instrument and DLS (panel B). The trace corresponds to particles on each of the lines in the image. The blue line highlights the smaller size population which range in size from 15 – 50 nm while the red trace is for the larger particles that were observed to be up to 400 nm in size.

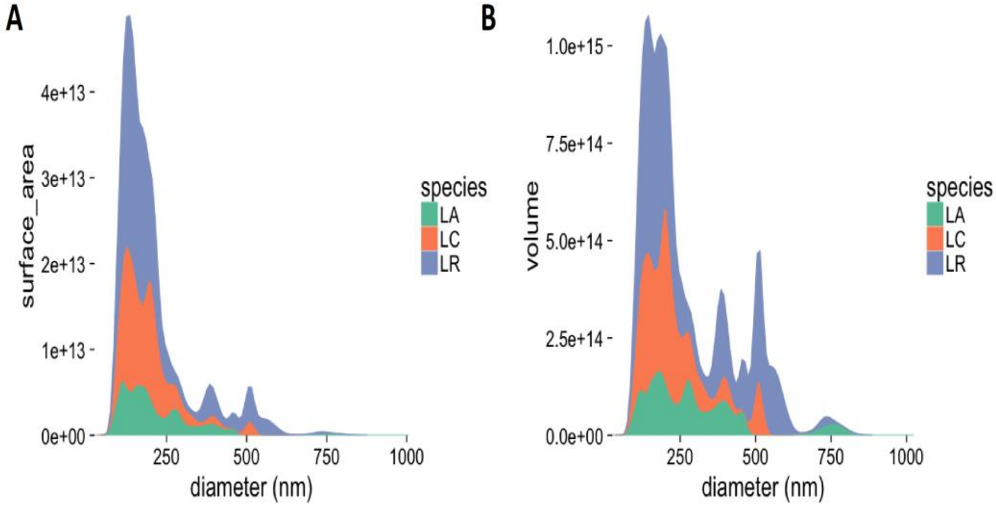

Fig. S4. Surface area (A) and volume (B) measurements of *L. acidophilus*, *L. casei*, and *L. reuteri* from NanoSight experiments. Purified MVs were diluted in 1:100 or 1:1000 in PBS. Measurements were performed in triplicate.

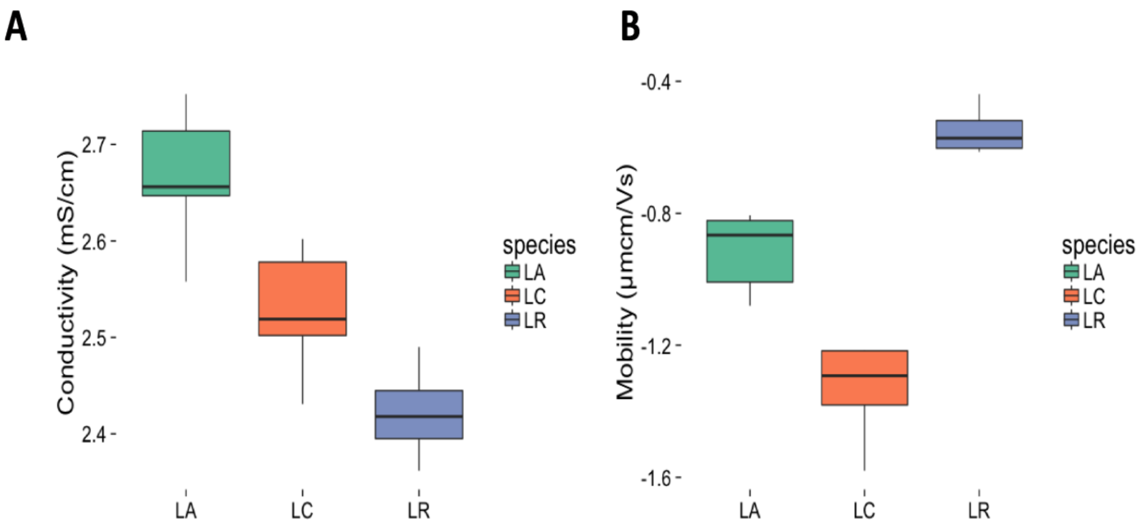

Fig. S5. Conductivity (A) and mobility (B) measurements of *L. acidophilus*, *L. casei*, and *L. reuteri* from DLS experiments. Purified MVs were diluted in 0.1 x PBS. Measurements were performed in triplicate.

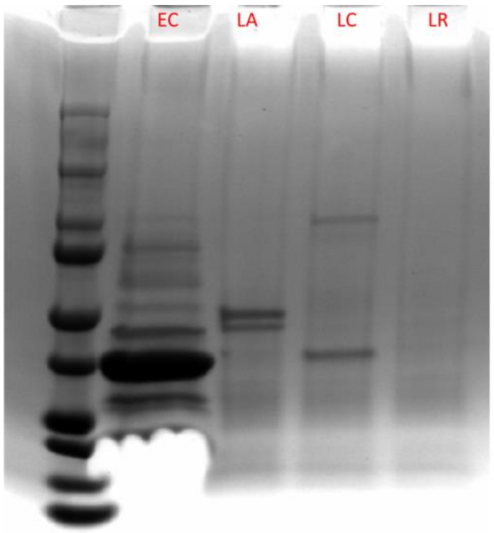

Fig. S6. SDS-PAGE of *Lactobacillus*-derived MV. Equivalent numbers of OMV/MV from *Escherichia coli* (EC), *L. acidophilus* (LA), *L. casei* (LC), and *L. reuteri* (LR) were resolved on 4-15% gradient polyacrylamide gels. Protein bands were visualized using GelCode staining reagent.

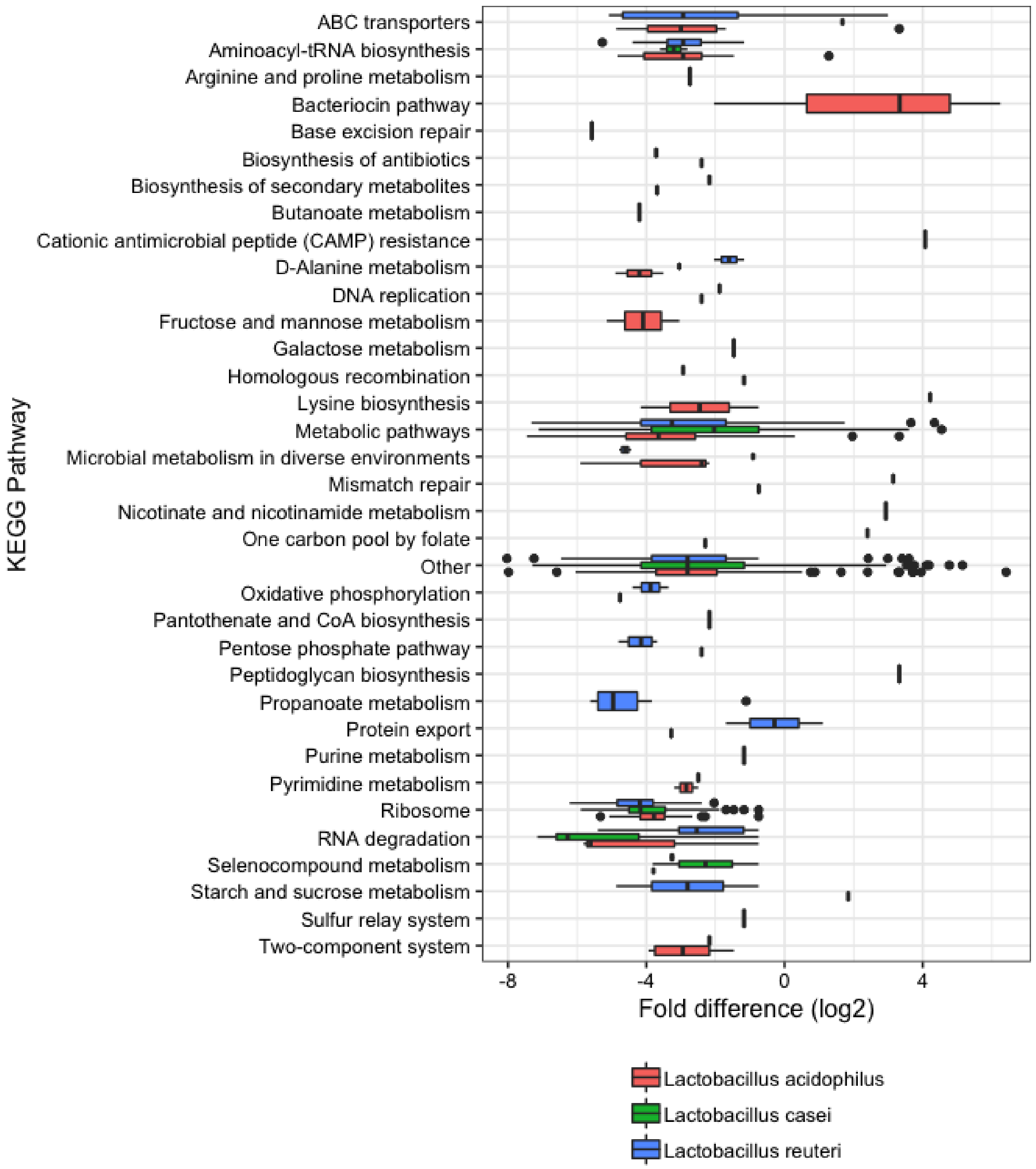

Fig. S7 Box plot clustering of proteomics for the *lactobacillus* species MVs. Data is clustered according the pathway/cellular function/role for each of the species examined.

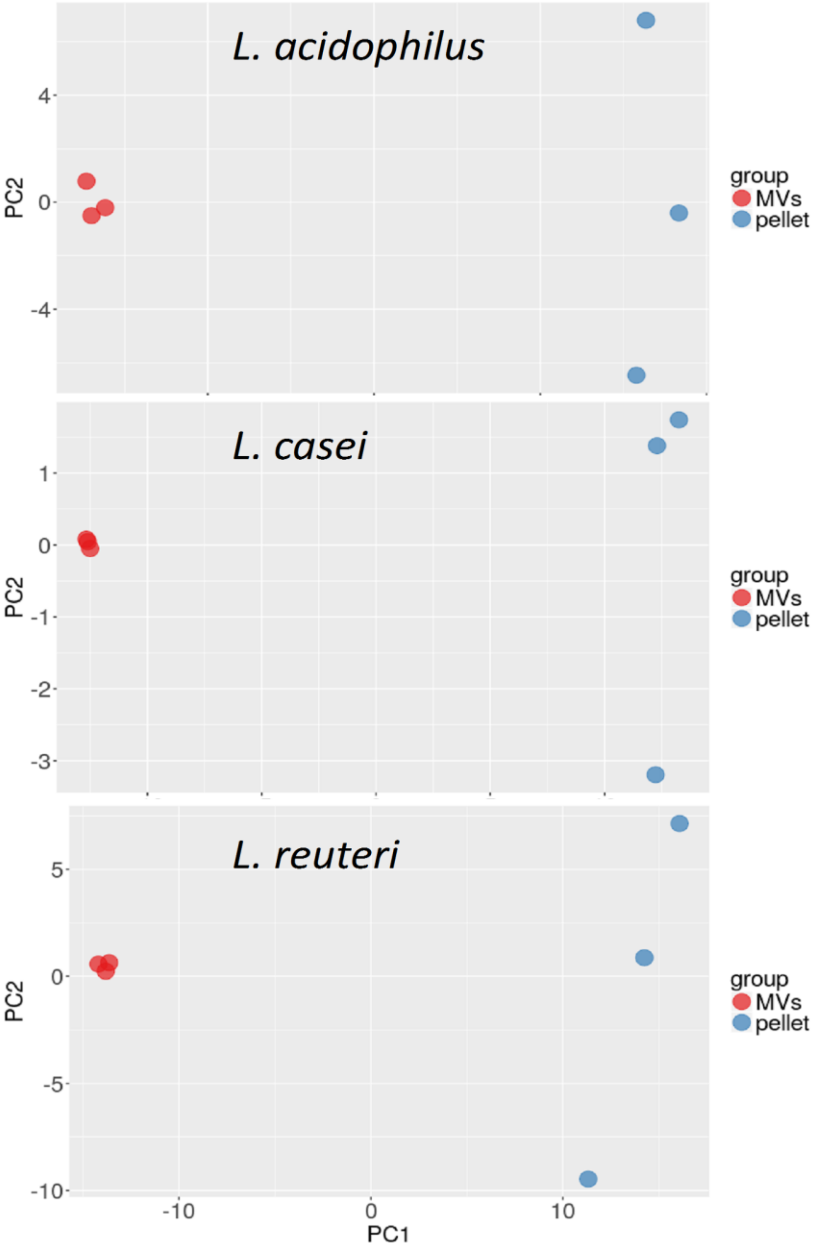

Fig. S8. Principal component analysis (PCA) on the proteomic data (n = 18) used in the study. PCAs were performed on normalized weighted spectral counts and grouped based on identification in the MVs or pellet.

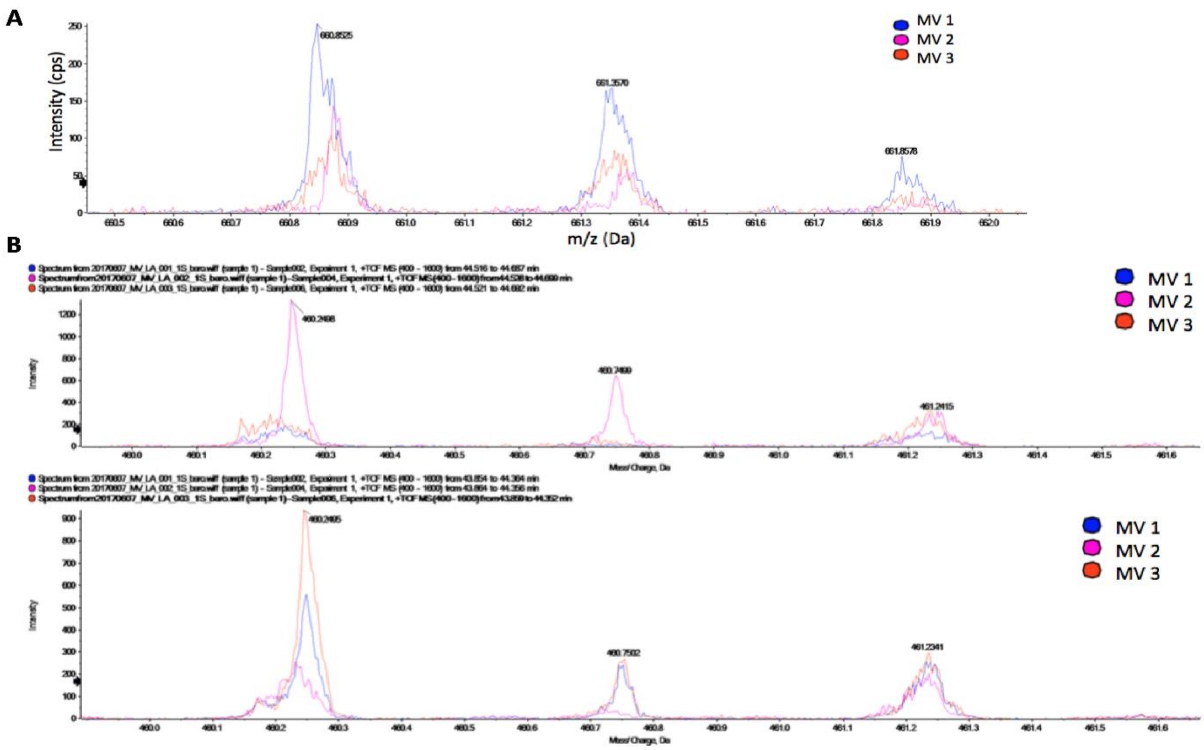

Fig. S9. (A) Precursor ion ((R)GLWENLSNIFK(H)) average MS spectra from retention time range 78.24-78.38 min. (B) Precursor ion ((K)APISGYVGR(G)) average MS spectra from retention time range 44.53-44.69 min (top) and 43.85-44.36 min (bottom), where the peptide in sample MV2 was eluted slightly later than in the samples MV1 and MV3.

Supplemental methods

Proteomics analysis. Triplicate biological samples of MVs and bacterial pellets from L. acidophilus, L. casei, and L. reuteri were harvested at 60 h. Pellets were lysed using OneShot (Constant Systems Ltd., Daventry, UK) at 40 kpsi pressure in 10% n-propanol in 50 mM ammonium bicarbonate (ABC) in a 10 mL suspension. The instrument was then washed with 10 mL ABC, the lysate and wash were combined, and then evaporated via speed-vac. Samples were normalized by total protein content to 100 µg prior digestion using the Pierce BCA Protein Assay Kit (Thermo Scientific, Rockford, IL). All samples were digested in solution with sequencing-grade modified trypsin (Promega, Madison, WI) at a 1:30 w/w enzyme to substrate ratio in a barocycler (Pressure Biosciences Inc., Easton, MA) for 90 min (90 cycles: 50 s on at 20 kpsi, 10 s off). Digested samples (150 µL) were evaporated via speed-vac. MVs were solubilized in 10% n-propanol, digested in solution and dried as described above for pellets. All dried samples were stored at -20 °C until they were analyzed by LC-MS/MS. Immediately prior to analysis, samples

were solubilized in solvent A (0.1% formic acid (FA) in HPLC grade water) and 10 µL of sample (~50 µg of total protein) was injected into the LC-MS/MS system (Tempo-MDLC coupled to a TripleTOF 5600 mass spectrometer - Sciex, Foster City, CA). Peptides were loaded for 15 min in 5% solvent B ((0.1% FA in acetonitrile) and 95% solvent A, separated on two eksigent C18 Chrom XP columns (150 x 0.3 mm, 120 A) connected in a row using a linear gradient of increasing mobile phase B in the rate of 0.52% per minute. The 180 min LC method also included 10 min column wash at 80% B and re-equilibration of the columns with the starting condition at 5% solvent B. Mass spectrometry method consisted of two Experiments - 1. TOF experiment analyzing precursor ions (400-1600 Da) and 2. information dependent MS/MS experiment set to monitor m/z range of 20-1600 Da. Seven highest precursor ions with intensities above 200 cps from Experiment 1 were submitted for analyses by Experiment 2. Precursor ions were excluded for 15 s after four repeated MS/MS experiments. Rolling collision energy was used for fragmentation. Analyst TF 1.7.1 was used as the acquisition software. Resulting MS/MS spectra were extracted by AB Sciex MS data convertor version 1.3 beta. Charge state deconvolution and deisotoping were not performed. All MS/MS samples were analyzed using Mascot (Matrix Science, London, UK; version 2.6.0) and X! Tandem (The GPM, thegpm.org; version CYCLONE (2010.12.01.1)). Mascot was set up to search the LacidoNCFM 000 (1,859 sequences; 583,994 residues), LreuteDSM 000 (1,865 sequences; 559,402 residues), LactoCasei (152,421 sequences; 44,664,331 residues), respectively and 2016_Contams_STDs_0000 database (190 sequences; 54,899 residues) assuming the digestion enzyme trypsin. X! Tandem was set up to search a reverse concatenated subset of the LacidoNCFM_000 database which contained only proteins identified by Mascot in all analyzed samples.

Mascot and X! Tandem were searched with a fragment ion mass tolerance of 0.60 Da and a parent ion tolerance of 0.60 Da. Deamidation of asparagine and glutamine and oxidation of methionine were specified in Mascot as variable modifications. Glu->pyro-Glu of the n-terminus, ammonia-loss of the n-terminus, gln->pyro-Glu of the n-terminus, deamidated of asparagine and glutamine and oxidation of methionine were specified in X! Tandem as variable modifications. Scaffold (version Scaffold_4.7.3, Proteome Software Inc., Portland, OR) was used to validate MS/MS based peptide and protein identifications. Peptide identifications were accepted if they could be established at greater than 70.0% probability by the Peptide Prophet algorithm (46) with Scaffold delta-mass correction. Protein identifications were accepted if they could be established at greater than 90.0% probability and contained at least 2 identified peptides. Protein probabilities were assigned by the Protein Prophet algorithm (41). Proteins that contained similar peptides and could not be differentiated based on MS/MS analysis alone were grouped to satisfy the principles of parsimony. Quantitative analysis was done in Scaffold using weighted spectra as an input. Only spectra satisfying the probability settings were considered for the analysis (lower scoring matches and probabilities <5% were not included).

The Mascot database search protocol as output by Mascot:

[L. acidophilus:(1010 entries) (only "20170607_Pellet_LA_001_1S_baro (F007511)") also assuming trypsin, a reverse concatenated subset of the LacidoNCFM_000 database (1018 entries) (only "20170607_Pellet_LA_003_1S_baro (F007509)") also assuming trypsin, a reverse concatenated subset of the LacidoNCFM_000 database (1026 entries) (only "20170607_Pellet_LA_002_1S_baro (F007510)") also assuming a reverse concatenated subset of the LacidoNCFM_000 database (1044 entries) (only "20170607_MV_LA_001_1S_baro

(F007508)") also assuming trypsin, a reverse concatenated subset of the LacidoNCFM_000 database (578 entries) (only "20170607_MV_LA_002_1S_baro (F007507)") also assuming trypsin and a reverse concatenated subset of the LacidoNCFM_000 database (664 entries) (only "20170607_MV_LA_003_1S_baro (F007506)")

L. Reuterii: X! Tandem was set up to search a reverse concatenated subset of the LreuteDSM_000 database (1040 entries) (only "20170612_Pellet_LR_002_1S_baro (F007513)") also assuming trypsin, a reverse concatenated subset of the LreuteDSM_000 database (unknown version, 1204 entries) (only "20170612_Pellet_LR_001_1S_baro (F007512)") also assuming trypsin, a reverse concatenated subset of the LreuteDSM_000 database (unknown version, 458 entries) (only "20170612_MV_LR_002_1S_baro (F007516)") also assuming trypsin, a reverse concatenated subset of the LreuteDSM_000 database (unknown version, 466 entries) (only "20170612_MV_LR_003_1S_baro (F007517)") also assuming trypsin, a reverse concatenated subset of the LreuteDSM_000 database (unknown version, 632 entries) (only "20170612_MV_LR_001_1S_baro (F007515)") also assuming trypsin and a reverse concatenated subset of the LreuteDSM_000 database (unknown version, 968 entries) (only "20170612_Pellet_LR_003_1S_baro (F007514)") also assuming trypsin. also assuming trypsin.

L. casei: X! Tandem was set up to search a reverse concatenated subset of the LactoCasei_sequence database (21012 entries) (only "20170609_Pellet_LC_003_1S_baro (F007482)") also assuming trypsin, a reverse concatenated subset of the LactoCasei_sequence database (unknown version, 22064 entries) (only "20170609_Pellet_LC_002_1S_baro (F007483)") also assuming trypsin, a reverse concatenated subset of the LactoCasei_sequence database (unknown version, 22076 entries) (only "20170609_Pellet_LC_001_1S_baro (F007484)") also assuming trypsin, a reverse concatenated subset of the LactoCasei_sequence database (unknown version, 2692 entries) (only "20170609_MV_LC_002_1S_baro (F007486)") also assuming trypsin, a reverse concatenated subset of the LactoCasei_sequence database (unknown version, 3602 entries) (only "20170609_MV_LC_003_1S_baro (F007485)") also assuming trypsin and a reverse concatenated subset of the LactoCasei_sequence database (unknown version, 4068 entries) (only "20170609_MV_LC_001_1S_baro (F007487)") also assuming trypsin

246
