# Supplementary material for: Isolation and characterization of Lactobacillus-derived membrane vesicles
Source: Sci Rep. 2019 Jan 29;9:877. doi: 10.1038/s41598-018-37120-6 (PMC6351534; doi:10.1038/s41598-018-37120-6)
Supplement: Supplementary file 2 — table s1 [file 41598_2018_37120_MOESM2_ESM.pdf]

Table S1. List of proteins identified in MVs and pellet *Lactobacillus acidophilus*.

Uniprot ID	Locus ID	MV sample 1	MV sample 2	MV sample 3	Pellet sample 1	Pellet sample 2	Pellet sample 3
SLAP_LACAC	slpA	639	355	604	89	96	125
Q5FL51_LACAC	LBA0698	591	400	593	325	468	378
Q5FJA7_LACAC	LBA1392	265	30	253	0	0	0
Q5FI65_LACAC	LBA1805	181	83	198	0	0	0
Q5FLN0_LACAC	slpX	84	98	165	5	3	4
Q5FM30_LACAC	LBA0352	84	53	99	36	34	40
Q5FI70_LACAC	LBA1800	121	0	99	3	2	2
Y378_LACAC	LBA0378	109	0	0	3	3	3
GLMU_LACAC	glmU	84	0	0	3	4	4
SYGB_LACAC	glyS	60	0	0	6	5	5
Q5FJ08_LACAC	LBA1497	24	0	33	15	10	15
Q5FIP8_LACAC	fmtB	24	0	22	0	0	0
Q5FKV1_LACAC	ftsQ	36	0	0	0	0	0
Q5FIG5_LACAC	LBA1697	36	0	0	0	0	0
Q5FMN9_LACAC	glnP	36	0	0	0	0	0
Q5FHZ8_LACAC	LBA1876	36	0	0	0	0	0
Q5FL54_LACAC	LBA0695	36	0	0	16	12	12
GATB_LACAC	gatB	24	0	0	29	25	27
Q5FKT1_LACAC	bipA	24	0	0	24	24	20
Q5FKG7_LACAC	LBA0955	24	0	0	0	0	0
Q5FKA5_LACAC	LBA1020	24	0	0	0	0	0

Q5FI08_LACAC	LBA1866	24	0	0	0	0	0
Q5FKV5_LACAC	LBA0805	24	0	0	0	0	0
Q5FI34_LACAC	LBA1838	24	0	0	0	0	0
GRPE_LACAC	grpE	24	0	0	6	2	6
Q5FM98_LACAC	clpC	0	23	0	2	4	3
EFG_LACAC	fusA	0	0	0	72	71	70
Q5FKF5_LACAC	LBA0968	0	0	0	45	46	46
Q5FHQ7_LACAC	poxB	0	0	0	77	75	75
Q5FHW6_LACAC	clpE	0	0	0	214	221	219
RL16_LACAC	rplP	0	0	0	9	9	10
Q5FI52_LACAC	LBA1820	0	0	0	14	14	14
Q5FIY9_LACAC	LBA1516	0	0	0	10	10	10
TPIS_LACAC	tpiA	0	0	0	56	59	57
Q5FLZ5_LACAC	LBA0388	0	0	0	16	17	17
RL9_LACAC	rplI	0	0	0	14	14	14
Q5FLF9_LACAC	LBA0585	0	0	0	20	21	21
Q5FKG5_LACAC	kpyK	0	0	0	187	181	177
CH10_LACAC	groS	0	0	0	19	19	20
Q5FIM1_LACAC	LBA1641	0	0	0	63	65	61
TIG_LACAC	tig	0	0	0	96	94	100
RL10_LACAC	rplJ	0	0	0	19	18	19
RL21_LACAC	rplU	0	0	0	6	6	6
Q5FIG3_LACAC	exoA	0	0	0	77	82	77

SYR_LACAC	argS	0	0	0	3	3	3
PTH_LACAC	pth	0	0	0	2	2	2
SYT_LACAC	thrS	0	0	0	39	37	36
SYL_LACAC	leuS	0	0	0	4	4	4
Q5FIA0_LACAC	pepF	0	0	0	2	2	2
Q5FK82_LACAC	LBA1046	0	0	0	1	1	1
G6PI_LACAC	pgi	0	0	0	84	80	86
RL31B_LACAC	rpmE2	0	0	0	10	9	10
Q5FIY8_LACAC	greA	0	0	0	16	16	17
Q5FJM6_LACAC	LBA1267	0	0	0	13	13	14
Q5FJ98_LACAC	LBA1401	0	0	0	13	12	12
RTPR_LACAC	rtpR	0	0	0	87	88	81
GLMM_LACAC	glmM	0	0	0	13	12	12
Q5FKK0_LACAC	citF	0	0	0	13	12	12
RL22_LACAC	rplV	0	0	0	12	11	11
SYP_LACAC	proS	0	0	0	22	22	20
Q5FMF7_LACAC	LBA0222	0	0	0	27	30	30
Q5FL15_LACAC	ackB	0	0	0	7	7	7
EFP1_LACAC	efp1	0	0	0	9	9	10
Q5FMP2_LACAC	LBA0131	0	0	0	20	20	18
GPMA_LACAC	gpmA	0	0	0	82	91	84
Q5FKC3_LACAC	LBA1001	0	0	0	10	9	9
RS10_LACAC	rpsJ	0	0	0	12	11	12

EFTS_LACAC	tsf	0	0	0	26	29	29
Q5FLA7_LACAC	clpE	0	0	0	8	7	8
Q5FLQ3_LACAC	LBA0486	0	0	0	7	7	8
Q5FJU8_LACAC	pepT	0	0	0	5	6	5
CH60_LACAC	groL	0	0	0	107	96	97
LDH2_LACAC	ldh2	0	0	0	9	8	9
RL13_LACAC	rplM	0	0	0	6	7	7
Q5FJI5_LACAC	LBA1310	0	0	0	20	19	22
RL19_LACAC	rplS	0	0	0	11	11	12
Q5FKI3_LACAC	LBA0937	0	0	0	17	15	15
PGK_LACAC	pgk	0	0	0	140	143	158
Q5FME3_LACAC	LBA0236	0	0	0	10	12	10
Q5FLA5_LACAC	ptsI	0	0	0	55	50	56
Q5FI89_LACAC	fruK	0	0	0	54	47	52
RL6_LACAC	rplF	0	0	0	47	41	46
ATPB_LACAC	atpD	0	0	0	37	36	33
PYRH_LACAC	pyrH	0	0	0	12	12	10
Q5FJJ4_LACAC	oppA	0	0	0	35	32	30
Q5FN08_LACAC	LBA0008	0	0	0	3	3	3
GLYA_LACAC	glyA	0	0	0	13	15	14
PDRP_LACAC	LBA1206	0	0	0	16	16	14
Q5FKU9_LACAC	ftsZ	0	0	0	16	15	14
Q5FHQ8_LACAC	LBA1973	0	0	0	27	30	31

Q5FI39_LACAC	LBA1833	0	0	0	9	9	8
Q5FLF7_LACAC	rbsK	0	0	0	14	15	16
Q5FIZ4_LACAC	LBA1511	0	0	0	16	17	18
Q5FHV1_LACAC	dltA	0	0	0	40	37	42
RL5_LACAC	rplE	0	0	0	26	30	29
RL20_LACAC	rplT	0	0	0	13	12	12
RL27_LACAC	rpmA	0	0	0	9	9	10
Q5FJX9_LACAC	LBA1156	0	0	0	4	5	5
Q5FMH4_LACAC	LBA0205	0	0	0	43	48	50
DDL_LACAC	ddl	0	0	0	32	33	29
Q5FLX0_LACAC	zwf	0	0	0	18	18	16
Q5FKM5_LACAC	LBA0890	0	0	0	8	9	10
MURC_LACAC	murC	0	0	0	22	19	19
Q5FMW5_LACAC	ldhD	0	0	0	121	104	106
Q5FN14_LACAC	dnaN	0	0	0	6	6	5
Y733_LACAC	LBA0733	0	0	0	6	7	7
Q5FJ73_LACAC	LBA1426	0	0	0	6	7	7
Q5FLA6_LACAC	ptsH	0	0	0	41	36	43
Q5FIK6_LACAC	LBA1656	0	0	0	18	17	15
EFP2_LACAC	efp2	0	0	0	14	12	14
EFTU_LACAC	tuf	0	0	0	794	948	897
RS2_LACAC	rpsB	0	0	0	38	32	33
Q5FJG3_LACAC	LBA1334	0	0	0	11	10	9

DNAK_LACAC	dnaK	0	0	0	82	75	90
Q5FIJ7_LACAC	oppA	0	0	0	42	39	35
Q5FI23_LACAC	pepN	0	0	0	47	44	39
Q5FLK4_LACAC	LBA0539	0	0	0	6	5	5
Q5FMX9_LACAC	LBA0040	0	0	0	13	16	15
SYE_LACAC	gltX	0	0	0	12	12	10
KAD_LACAC	adk	0	0	0	26	29	24
RL18_LACAC	rplR	0	0	0	42	35	38
RPOA_LACAC	rpoA	0	0	0	13	15	13
Q5FLW8_LACAC	LBA0415	0	0	0	39	40	34
Q5FLK9_LACAC	LBA0534	0	0	0	16	13	14
Q5FI90_LACAC	fruA	0	0	0	42	46	38
Q5FI97_LACAC	LBA1769	0	0	0	29	24	25
Q5FLE5_LACAC	LBA0600	0	0	0	14	13	12
RPOB_LACAC	rpoB	0	0	0	32	29	27
SYY_LACAC	tyrS	0	0	0	5	6	7
Q5FN03_LACAC	LBA0014	0	0	0	20	18	22
Q5FL76_LACAC	yvyD	0	0	0	35	29	35
RS3_LACAC	rpsC	0	0	0	13	13	11
RS12_LACAC	rpsL	0	0	0	15	12	13
RS6_LACAC	rpsF	0	0	0	15	17	19
Q5FMM2_LACAC	LBA0151	0	0	0	8	6	7
Q5FMY4_LACAC	LBA0035	0	0	0	3	3	3

PFKA_LACAC	pfkA	0	0	0	42	52	48
Q5FKQ7_LACAC	LBA0856	0	0	0	17	14	14
RL23_LACAC	rplW	0	0	0	10	11	12
Q5FIS9_LACAC	LBA1578	0	0	0	19	17	21
Q5FM39_LACAC	LBA0343	0	0	0	10	10	12
RS7_LACAC	rpsG	0	0	0	41	50	51
Q5FJF6_LACAC	ilvE	0	0	0	8	10	10
Q5FMM8_LACAC	LBA0145	0	0	0	8	7	6
Q5FJX3_LACAC	LBA1162	0	0	0	24	22	19
Q5FLY7_LACAC	LBA0396	0	0	0	63	53	51
ATPF_LACAC	atpF	0	0	0	11	12	9
RL15_LACAC	rplO	0	0	0	9	7	7
GUAA_LACAC	guaA	0	0	0	16	16	13
ACP_LACAC	acpP	0	0	0	4	4	5
GATC_LACAC	gatC	0	0	0	6	6	7
Q5FKW1_LACAC	mreB	0	0	0	32	27	27
Q5FL45_LACAC	LBA0704	0	0	0	13	13	15
LUXS_LACAC	luxS	0	0	0	5	6	5
IF2_LACAC	infB	0	0	0	32	25	29
PPAC_LACAC	ppaC	0	0	0	38	30	36
RL1_LACAC	rplA	0	0	0	27	25	31
RL17_LACAC	rplQ	0	0	0	17	21	20
Q5FKK3_LACAC	citC	0	0	0	13	14	11

Q5FLP2_LACAC	LBA0497	0	0	0	10	13	13
Q5FIE2_LACAC (+1)	LBA1720	0	0	0	8	10	10
RPOC_LACAC	rpoC	0	0	0	29	32	26
MURD_LACAC	murD	0	0	0	8	10	9
RS8_LACAC	rpsH	0	0	0	19	15	18
Q5FLS6_LACAC	adhE	0	0	0	8	7	9
UPP_LACAC	upp	0	0	0	22	19	18
ATPG_LACAC	atpG	0	0	0	3	4	4
Q5FLT1_LACAC	manN	0	0	0	2	2	2
Q5FI48_LACAC	ychF	0	0	0	19	17	15
SYI_LACAC	ileS	0	0	0	2	3	2
PURA_LACAC	purA	0	0	0	24	25	19
Q5FLC0_LACAC	LBA0625	0	0	0	32	32	26
Q5FMI7_LACAC	LBA0191	0	0	0	24	23	18
ERA_LACAC	era	0	0	0	19	15	17
GATA_LACAC	gatA	0	0	0	11	8	10
DLTC_LACAC	dltC	0	0	0	11	8	10
RL2_LACAC	rplB	0	0	0	32	25	30
SYC_LACAC	cysS	0	0	0	6	8	8
Q5FJ04_LACAC	glnA	0	0	0	53	63	70
Q5FIR0_LACAC	fbaA	0	0	0	126	155	167
Q5FKF3_LACAC	LBA0970	0	0	0	108	128	144
RL11_LACAC	rplK	0	0	0	21	16	17

Q5FKQ1_LACAC	LBA0864	0	0	0	22	17	18
Q5FJB1_LACAC	LBA1388	0	0	0	66	66	50
Q5FMA0_LACAC	lysS	0	0	0	21	22	16
Q5FKM3_LACAC	bshA	0	0	0	5	5	4
Q5FM58_LACAC	rpsI	0	0	0	16	12	16
LDH1_LACAC	ldh1	0	0	0	47	63	55
RS5_LACAC	rpsE	0	0	0	14	15	11
Q5FKH2_LACAC	LBA0950	0	0	0	3	4	4
RS16_LACAC	rpsP	0	0	0	9	9	12
RS11_LACAC	rpsK	0	0	0	13	17	13
RL29_LACAC	rpmC	0	0	0	11	8	11
Q5FMF0_LACAC	LBA0229	0	0	0	8	8	6
Q5FI30_LACAC	LBA1842	0	0	0	13	11	9
Q5FKH9_LACAC	LBA0942	0	0	0	23	19	17
Q5FMS3_LACAC	LBA0100	0	0	0	9	7	9
Q5FMF6_LACAC	cdpA	0	0	0	16	12	14
Q5FIU1_LACAC	LBA1566	0	0	0	9	7	7
Q5FMT9_LACAC	htrA	0	0	0	13	10	9
Q5FK98_LACAC	LBA1027	0	0	0	5	4	4
Q5FMH5_LACAC	pepE	0	0	0	16	12	12
Q5FM46_LACAC	LBA0336	0	0	0	8	11	11
FMT_LACAC	fmt	0	0	0	2	3	3
Q5FMM4_LACAC	uspA	0	0	0	7	8	6

Q5FMY1_LACAC	LBA0038	0	0	0	4	3	4
Q5FJK1_LACAC	LBA1293	0	0	0	5	6	8
KITH_LACAC	tdk	0	0	0	4	5	4
Y1592_LACAC	LBA1592	0	0	0	23	17	24
Q5FIY4_LACAC	LBA1521	0	0	0	5	6	4
RS21_LACAC	rpsU	0	0	0	3	4	5
Q5FK97_LACAC	LBA1029	0	0	0	3	3	4
ATPA_LACAC	atpA	0	0	0	40	35	28
Q5FMI0_LACAC	LBA0199	0	0	0	22	26	18
MURE_LACAC	murE	0	0	0	19	22	16
Q5FML4_LACAC	LBA0159	0	0	0	9	8	6
Q5FHV8_LACAC	LBA1919	0	0	0	3	4	3
Q5FK83_LACAC	LBA1045	0	0	0	10	8	8
Q5FJK0_LACAC	LBA1294	0	0	0	13	9	10
Q5FIM0_LACAC	LBA1642	0	0	0	7	5	5
PYRG_LACAC	pyrG	0	0	0	30	39	28
Q5FK81_LACAC	LBA1047	0	0	0	4	4	3
Y418_LACAC	LBA0418	0	0	0	12	10	14
Q5FLV4_LACAC	LBA0429	0	0	0	13	15	18
Q5FJQ3_LACAC	LBA1238	0	0	0	3	2	3
RL4_LACAC	rplD	0	0	0	22	17	15
Q5FIS0_LACAC	prtM	0	0	0	20	14	15
SYA_LACAC	alaS	0	0	0	14	20	15

Q5FKD6_LACAC	LBA0987	0	0	0	37	40	53
Q5FIL6_LACAC	LBA1646	0	0	0	6	6	4
RS17_LACAC	rpsQ	0	0	0	15	11	16
IF1_LACAC	infA	0	0	0	5	4	5
TSAD_LACAC	tsaD	0	0	0	8	6	5
Q5FKD0_LACAC	pepD	0	0	0	17	17	12
Q5FI38_LACAC	LBA1834	0	0	0	7	10	8
ENO_LACAC	eno	0	0	0	399	560	602
Q5FMI4_LACAC	pepG	0	0	0	5	7	6
TYSY_LACAC	thyA	0	0	0	4	4	3
ATPD_LACAC	atpH	0	0	0	5	5	4
Q5FKX5_LACAC	LBA0783	0	0	0	16	19	12
Q5FLM1_LACAC	nadE	0	0	0	8	5	6
RL30_LACAC	rpmD	0	0	0	8	7	10
CAPP_LACAC	ppc	0	0	0	19	20	13
Q5FM15_LACAC	LBA0367	0	0	0	5	5	4
GPDA_LACAC	gpsA	0	0	0	12	14	9
APT_LACAC	apt	0	0	0	10	6	8
Q5FJI4_LACAC	LBA1311	0	0	0	10	7	7
RS4_LACAC	rpsD	0	0	0	24	19	30
Q5FI56_LACAC	LBA1816	0	0	0	7	4	6
Q5FMF5_LACAC	prsA	0	0	0	21	16	13
Q5FIH7_LACAC	LBA1685	0	0	0	6	9	7

RPOZ_LACAC	rpoZ	0	0	0	5	4	3
Q5FKT2_LACAC	def	0	0	0	5	4	3
RL3_LACAC	rplC	0	0	0	19	17	27
Q5FJT4_LACAC	LBA1204	0	0	0	4	5	6
Q5FJQ6_LACAC	LBA1235	0	0	0	9	6	9
Q5FM24_LACAC	nusG	0	0	0	8	5	8
RS20_LACAC	rpsT	0	0	0	7	7	10
Q5FM32_LACAC	LBA0350	0	0	0	5	6	4
Q5FMN5_LACAC	LBA0138	0	0	0	3	3	2
Q5FKK1_LACAC	citE	0	0	0	19	13	13
Q5FII2_LACAC	LBA1680	0	0	0	3	5	3
Q5FLS5_LACAC	glmS	0	0	0	14	8	11
Q5FJT9_LACAC	glyQ	0	0	0	5	7	4
Q5FIB9_LACAC	LBA1744	0	0	0	4	3	4
RL14_LACAC	rplN	0	0	0	6	5	4
HSLU_LACAC	hslU	0	0	0	8	9	5
Q5FL96_LACAC	LBA0649	0	0	0	15	9	12
Q5FML7_LACAC	LBA0156	0	0	0	11	7	7
FTHS1_LACAC	fhs1	0	0	0	10	7	6
Q5FLV2_LACAC	ccpA	0	0	0	12	13	7
Q5FLW1_LACAC	LBA0422	0	0	0	36	22	23
Q5FL16_LACAC	LBA0740	0	0	0	2	1	1
RF1_LACAC	prfA	0	0	0	10	6	6

RS18_LACAC	rpsR	0	0	0	6	4	7
Q5FMB1_LACAC	LBA0270	0	0	0	4	2	3
Q5FIU0_LACAC	LBA1567	0	0	0	9	5	6
Q5FL92_LACAC	pthA	0	0	0	7	4	7
Q5FMM9_LACAC	LBA0144	0	0	0	3	2	4
Q5FI44_LACAC	parB	0	0	0	8	4	7
Q5FMA3_LACAC	ftsH	0	0	0	6	5	3
Q5FIW7_LACAC	infC	0	0	0	6	3	4
SYD_LACAC	aspS	0	0	0	2	3	4
Q5FK32_LACAC	LBA1098	0	0	0	3	4	2
OBG_LACAC	obg	0	0	0	2	2	1
Q5FMK8_LACAC	pepO	0	0	0	2	3	2
Q5FMK6_LACAC	LBA0167	0	0	0	7	4	9
Q5FKP7_LACAC	LBA0868	0	0	0	3	5	7
Q5FIK4_LACAC	LBA1658	0	0	0	5	5	3
Q5FN01_LACAC	LBA0016	0	0	0	8	5	4
CLPP_LACAC	clpP	0	0	0	10	12	6
RS13_LACAC	rpsM	0	0	0	10	6	13
Q5FMG6_LACAC	metG	0	0	0	15	16	8
Q5FMJ9_LACAC	LBA0176	0	0	0	3	3	2
Q5FLS2_LACAC	mutT	0	0	0	5	3	4
RL7_LACAC	rplL	0	0	0	76	35	73
Q5FHY5_LACAC	purB	0	0	0	7	13	16

Q5FIX9_LACAC	LBA1526	0	0	0	3	2	2
Q5FLT3_LACAC	manL	0	0	0	5	2	5
DEOC_LACAC	deoC	0	0	0	3	4	2
Q5FIS6_LACAC	LBA1581	0	0	0	7	7	13
Q5FJN3_LACAC	nusA	0	0	0	4	2	5
Q5FHU8_LACAC	LBA1929	0	0	0	1	3	3
Q5FJK4_LACAC	ffh	0	0	0	11	5	7
RS19_LACAC	rpsS	0	0	0	21	12	29
Q5FIT9_LACAC	LBA1568	0	0	0	2	2	4
Q5FIL7_LACAC	LBA1645	0	0	0	7	3	4
Q5FLM2_LACAC	nadC	0	0	0	1	2	1
Q5FL62_LACAC	LBA0687	0	0	0	4	8	4
Q5FL69_LACAC	trxB	0	0	0	2	5	3
Q5FN02_LACAC	LBA0015	0	0	0	3	6	3
Q5FLW7_LACAC	LBA0416	0	0	0	2	4	2
Q5FI60_LACAC	LBA1812	0	0	0	5	4	2
ACPS_LACAC	acpS	0	0	0	3	3	1
Q5FJT5_LACAC	phoH	0	0	0	8	5	3
Q5FIQ0_LACAC	LBA1609	0	0	0	3	3	6
DER_LACAC	der	0	0	0	2	1	1
SYV_LACAC	valS	0	0	0	4	4	2
Q5FHR1_LACAC	LBA1970	0	0	0	2	1	3
Q5FIM9_LACAC	ssdh	0	0	0	3	1	2

Q5FJG4_LACAC	nusB	0	0	0	2	2	4
Q5FLV5_LACAC	LBA0428	0	0	0	4	2	3
Q5FJN4_LACAC	LBA1258	0	0	0	3	1	4
Q5FJI9_LACAC	oppD	0	0	0	5	5	2
Q5FKB8_LACAC	LBA1006	0	0	0	3	1	2
Q5FLD2_LACAC	hprT	0	0	0	2	1	3
Q5FMH0_LACAC	LBA0209	0	0	0	8	6	2
Q5FIC4_LACAC	hflX	0	0	0	2	4	1
Q5FLY8_LACAC	frc	0	0	0	3	8	3
Q5FIU3_LACAC	LBA1564	0	0	0	8	3	3
RL28_LACAC	rpmB	0	0	0	2	1	5
Q5FI72_LACAC	LBA1798	0	0	0	4	4	0
Q5FLG5_LACAC	glnQ	0	0	0	2	2	0
Q5FI35_LACAC	LBA1837	0	0	0	1	1	0
Q5FKV0_LACAC	ftsA	0	0	0	2	0	2
RL32_LACAC	rpmF	0	0	0	4	5	0
Q5FKM8_LACAC	LBA0887	0	0	0	4	3	0
CITD_LACAC	citD	0	0	0	3	0	4
Q5FMR5_LACAC	LBA0108	0	0	0	3	3	0
Q5FHV4_LACAC	dltD	0	0	0	3	4	0
Q5FJL9_LACAC	pepO	0	0	0	5	4	0
Q5FIY0_LACAC	lisR	0	0	0	8	6	0
Q5FKK9_LACAC	LBA0908	0	0	0	1	2	0

Q5FL44_LACAC	LBA0705	0	0	0	2	0	2
Q5FKD9_LACAC	hslV	0	0	0	0	4	3
Q5FKX8_LACAC	mreB	0	0	0	3	0	4
Q5FIY3_LACAC	acyP	0	0	0	4	0	3
SYFB_LACAC	pheT	0	0	0	0	2	1
Q5FME4_LACAC	LBA0235	0	0	0	5	3	0
Q5FK76_LACAC	LBA1052	0	0	0	3	0	2
Q5FJC6_LACAC	pepX	0	0	0	2	0	4
Q5FM26_LACAC	LBA0356	0	0	0	2	0	3
Q5FKY9_LACAC	hemK	0	0	0	5	0	3
Q5FN10_LACAC	gyrA	0	0	0	0	3	1
Q5FIQ2_LACAC	LBA1607	0	0	0	3	0	1
Q5FI73_LACAC	LBA1797	0	0	0	4	0	2
Q5FI94_LACAC	psaA	0	0	0	3	1	0
DNAJ_LACAC	dnaJ	0	0	0	1	4	0
Q5FLV3_LACAC	LBA0430	0	0	0	5	0	1
Q5FIX7_LACAC	rsfS	0	0	0	3	0	0
Q5FHV2_LACAC	dltB	0	0	0	0	3	0
RSMA_LACAC	rsmA	0	0	0	0	2	0
RL35_LACAC	rpmI	0	0	0	1	0	0
Q5FJV0_LACAC	LBA1188	0	0	0	0	0	3
Q5FML5_LACAC	asnH	0	0	0	1	0	0
Q5FLF8_LACAC	LBA0586	0	0	0	3	0	0

Q5FLA8_LACAC	LBA0637	0	0	0	0	0	2
Q5FJJ0_LACAC	oppF	0	0	0	0	3	0
RPIA_LACAC	rpiA	0	0	0	0	3	0
Q5FKI1_LACAC	LBA0940	0	0	0	1	0	0
EX7S_LACAC	xseB	0	0	0	0	0	1
Q5FLI8_LACAC	LBA0555	0	0	0	3	0	0
Q5FIW6_LACAC	LBA1539	0	0	0	0	1	0
Q5FMB5_LACAC	murF	0	0	0	0	1	0
Q5FLT5_LACAC	rnr	0	0	0	1	0	0
Q5FMW1_LACAC	pbpX	0	0	0	0	0	3
MURG_LACAC	murG	0	0	0	0	0	1
Q5FKI7_LACAC	relA	0	0	0	0	2	0
Q5FHR5_LACAC	copA	0	0	0	0	0	1
Q5FI45_LACAC	parA	0	0	0	0	1	0
Q5FIN0_LACAC	LBA1631	0	0	0	0	2	0
Q5FKQ0_LACAC	LBA0865	0	0	0	0	1	0
Q5FKL0_LACAC	fumC	0	0	0	1	0	0
Q5FK04_LACAC	LBA1126	0	0	0	0	0	1
KGUA_LACAC	gmk	0	0	0	0	0	2
Q5FLM4_LACAC	tagA	0	0	0	0	1	0
Q5FKZ2_LACAC	LBA0765	0	0	0	0	1	0
Q5FJR9_LACAC	LBA1220	0	0	0	0	0	3
Q5FKY8_LACAC	LBA0769	0	0	0	4	0	0

Q5FIU2_LACAC	LBA1565	0	0	0	0	0	2
Q5FJ35_LACAC	galE	0	0	0	0	0	2
Q5FJU2_LACAC	rpoD	0	0	0	0	0	2
Q5FK51_LACAC	bshB	0	0	0	3	0	0
RECA_LACAC	recA	0	0	0	0	0	2
Q5FKK6_LACAC	LBA0911	0	0	0	0	0	2
DGK2_LACAC	LBA1950	0	0	0	2	0	0
