# Supplementary material for: Isolation and characterization of Lactobacillus-derived membrane vesicles
Source: Sci Rep. 2019 Jan 29;9:877. doi: 10.1038/s41598-018-37120-6 (PMC6351534; doi:10.1038/s41598-018-37120-6)
Supplement: Supplementary file 3 — table s2 [file 41598_2018_37120_MOESM3_ESM.pdf]

Table S2. List of proteins identified in MVs and pellet *Lactobacillus casei*.

Uniprot ID	Locus ID	MV sample 1	MV sample 2	MV sample 3	Pellet sample 1	Pellet sample 2	Pellet sample 3
Q03AK7_LACP3	LSEI_0967	1109	619	840	538	562	586
Q03CD1_LACP3	LSEI_0281	974	640	601	8	9	12
Q038J0_LACP3	LSEI_1608	636	561	434	82	95	97
Q036Q4_LACP3	LSEI_2065	536	519	402	94	55	124
Q03D36_LACP3	LSEI_0020	266	222	183	32	41	38
Q03CH3_LACP3	LSEI_0236	230	122	111	4	2	6
Q03AI8_LACP3	LSEI_0988	185	159	115	13	12	16
S6CK93_LACCA	LBCZ_2692	216	95	76	0	0	0
S6C5N2_LACCA	LBCZ_0371	167	138	72	0	0	0
Q03CC3_LACP3	LSEI_0289	86	116	72	5	3	7
Q038X2_LACP3	LSEI_1472	131	16	96	13	6	7
Q036T8_LACP3	LSEI_2029	81	69	56	7	8	5
PAGL2_LACP3	LSEI_2684	72	58	64	13	12	4
Q03CH3_LACP3	LSEI_0236	54	37	40	0	0	0
Q03BH5_LACP3	LSEI_0603	81	11	24	0	0	0
TPIS_LACP3	LSEI_0969	59	5	52	105	95	171
S6C5N2_LACCA	LBCZ_0371	23	53	24	0	0	0
ATPG_LACP3	LSEI_1165	36	16	32	3	0	1
Q035T2_LACP3	LSEI_2296	54	5	4	3	2	0
IF2_LACP3	LSEI_1573	27	11	20	0	4	1
Q038R1_LACP3	LSEI_1536	36	0	16	0	0	0

Q039D0_LACP	LSEI_1414	14	5	20	28	17	29
F16PC_LACP3	LSEI_2045	18	11	8	0	0	0
Q034Q4_LACP3	LSEI_2597	0	21	16	123	114	142
GATA_LACP3	LSEI_1058	32	0	4	0	1	0
GATB_LACP3	LSEI_1059	14	0	20	0	1	0
DER_LACP3	LSEI_1380	9	0	24	0	0	0
GLGA_LACP3	LSEI_2037	0	32	0	0	0	1
CH10_LACP3	LSEI_2239	14	5	12	91	129	100
Q03BV1_LACP3	LSEI_0470	18	0	12	1	3	3
CCA_LACP3	LSEI_1384	9	21	0	0	0	0
RL7_LACP3	LSEI_2272	5	21	4	113	104	109
S6C674_LACCA	LBCZ_0621	9	16	4	0	4	2
Q039G3_LACP3	LSEI_1381	18	0	8	126	227	138
DPO3_LACP3	LSEI_1578	9	16	0	0	0	0
CINA_LACP3	LSEI_0922	5	16	4	0	0	0
Q037M8_LACP3	LSEI_1829	14	5	4	0	0	0
Q03CR3_LACP3	LSEI_0145	18	0	4	6	17	23
ATPA_LACP3	LSEI_1164	9	5	8	17	16	22
MUTL_LACP3	LSEI_2234	9	11	0	0	0	0
EFTU_LACP3	LSEI_1332	0	16	0	477	469	380
DEF_LACP3	LSEI_1303	0	16	0	0	0	0
GUAA_LACP3	LSEI_1979	0	11	4	0	4	2
Q034U9_LACP3	LSEI_2550	14	0	0	0	0	0

G6PI_LACP3	LSEI_1126	5	5	4	99	72	53
PURL_LACP3	LSEI_1751	9	0	4	0	0	0
FMT_LACP3	LSEI_1625	9	0	4	0	0	0
ERA_LACP3	LSEI_1515	9	0	4	0	1	0
FTHS_LACP3	LSEI_1460	0	11	0	0	0	0
PYRDA_LACP3	LSEI_1451	0	11	0	0	0	0
FOLD_LACP3	LSEI_1639	0	11	0	0	0	0
CH60_LACP3	LSEI_2238	5	5	0	379	420	353
NADE_LACP3	LSEI_1804	9	0	0	0	1	1
GLGC_LACP3	LSEI_2039	9	0	0	0	0	0
Q035V8_LACP3	LSEI_2270	9	0	0	0	0	0
ENO_LACP3	LSEI_0970	5	0	0	452	350	303
DNAK_LACP3	LSEI_1565	5	0	0	116	185	176
ATPB_LACP3	LSEI_1166	5	0	0	30	22	24
Q03CI4_LACP3	LSEI_0225	5	0	0	87	27	36
RS13_LACP3	LSEI_2479	5	0	0	0	22	4
DDL_LACP3	LSEI_0143	5	0	0	0	4	2
EFTS_LACP3	LSEI_1585	0	5	0	3	10	9
EFG_LACP3	LSEI_2508	0	5	0	2	12	6
Q039H9_LACP3	LSEI_1365	0	0	0	308	245	283
PGK_LACP3	LSEI_0968	0	0	0	279	166	239
Q036R9_LACP3	LSEI_2048	0	0	0	230	248	236
CH60_LACP3	LSEI_2238	0	0	0	209	232	216

Q033V8_LACP3	LSEI_2800	0	0	0	135	114	241
Q037U0_LACP3	LSEI_1760	0	0	0	140	221	146
Q03B97_LACP3	LSEI_0685	0	0	0	177	120	118
Q034V0_LACP3	LSEI_2549	0	0	0	125	110	111
Q039G5_LACP3	LSEI_1379	0	0	0	61	127	141
Q035B9_LACP3	LSEI_2467	0	0	0	114	63	129
TIG_LACP3	LSEI_1338	0	0	0	84	95	61
Q03B95_LACP3	LSEI_0687	0	0	0	99	73	75
Q036I8_LACP3	LSEI_2134	0	0	0	70	72	78
RL5_LACP3	LSEI_2491	0	0	0	62	49	68
Q03A86_LACP3	LSEI_1093	0	0	0	56	50	69
Q036K4_LACP3	LSEI_2118	0	0	0	55	54	66
RL11_LACP3	LSEI_2280	0	0	0	59	33	48
RS2_LACP3	LSEI_1586	0	0	0	53	53	58
RL18_LACP3	LSEI_2487	0	0	0	52	55	61
Q037U1_LACP3	LSEI_1759	0	0	0	46	50	42
RL10_LACP3	LSEI_2273	0	0	0	51	46	38
RL2_LACP3	LSEI_2500	0	0	0	35	52	43
ENO_LACP3	LSEI_0970	0	0	0	38	46	51
Q036B1_LACP3	LSEI_2214	0	0	0	36	41	49
Q03AN1_LACP3	LSEI_0942	0	0	0	35	38	35
RS8_LACP3	LSEI_2489	0	0	0	28	43	26
RS7_LACP3	LSEI_2509	0	0	0	28	21	40

Q03BY4_LACP3	LSEI_0432	0	0	0	41	24	28
Q033T0_LACP3	LSEI_2829	0	0	0	36	28	29
Q038B8_LACP3	LSEI_1681	0	0	0	24	23	26
RL24_LACP3	LSEI_2492	0	0	0	22	20	35
RL1_LACP3	LSEI_2279	0	0	0	31	23	23
RL3_LACP3	LSEI_2503	0	0	0	19	33	20
RL29_LACP3	LSEI_2495	0	0	0	19	18	24
Q034S6_LACP3	LSEI_2573	0	0	0	22	22	17
GPSB_LACP3	LSEI_1478	0	0	0	16	19	27
RPOA_LACP3	LSEI_2477	0	0	0	11	22	26
DNAK_LACP3	LSEI_1565	0	0	0	15	29	15
PFKA_LACP3	LSEI_1364	0	0	0	25	17	16
RL4_LACP3	LSEI_2502	0	0	0	19	19	19
Q03BE5_LACP3	LSEI_0636	0	0	0	13	25	17
Q03BZ6_LACP3	LSEI_0420	0	0	0	10	21	26
RL23_LACP3	LSEI_2501	0	0	0	11	13	17
RL22_LACP3	LSEI_2498	0	0	0	19	17	15
RL22_LACP3	LSEI_2498	0	0	0	10	19	24
RS16_LACP3	LSEI_1601	0	0	0	16	17	18
Q037Y5_LACP3	LSEI_1714	0	0	0	15	13	18
Q03BE0_LACP3	LSEI_0641	0	0	0	13	16	20
Q035A2_LACP3	LSEI_2484	0	0	0	17	13	18
DLTC_LACP3	LSEI_0796	0	0	0	12	16	18

Q03AM4_LACP3	LSEI_0949	0	0	0	8	16	20
Q038N9_LACP3	LSEI_1559	0	0	0	17	9	15
Y1724_LACP3	LSEI_1724	0	0	0	12	13	15
Q039N5_LACP3	LSEI_1306	0	0	0	13	12	12
Q03A40_LACP3	LSEI_1142	0	0	0	15	8	10
SYT_LACP3	LSEI_1703	0	0	0	8	11	14
Q034P8_LACP3	LSEI_2603	0	0	0	7	16	16
Q037P4_LACP3	LSEI_1808	0	0	0	11	14	11
RL20_LACP3	LSEI_1697	0	0	0	10	12	17
Q034Q4_LACP3	LSEI_2597	0	0	0	9	16	13
Q03C19_LACP3	LSEI_0397	0	0	0	10	9	10
Q039F6_LACP3	LSEI_1388	0	0	0	11	8	11
Q03CJ0_LACP3	LSEI_0219	0	0	0	9	6	9
S6CJH9_LACCA	LBCZ_1372	0	0	0	7	11	8
CLPP_LACP3	LSEI_0963	0	0	0	6	8	7
Y1022_LACP3	LSEI_1022	0	0	0	5	9	6
S6C9V3_LACCA	LBCZ_0884	0	0	0	5	5	2
RPOB_LACP3	LSEI_2516	0	0	0	4	7	6
SYE_LACP3	LSEI_2313	0	0	0	7	4	8
Q035W3_LACP3	LSEI_2265	0	0	0	3	8	6
Q03AL8_LACP3	LSEI_0955	0	0	0	5	5	6
Q038F2_LACP3	LSEI_1646	0	0	0	4	4	7
Q034W7_LACP3	LSEI_2532	0	0	0	5	4	5

Q039M8_LACP3	LSEI_1313	0	0	0	3	5	3
Q035A4_LACP3	LSEI_2482	0	0	0	4	4	3
ATPF_LACP3	LSEI_1162	0	0	0	4	4	5
Q033T2_LACP3	LSEI_2827	0	0	0	6	3	3
Q039T3_LACP3	LSEI_1255	0	0	0	4	2	2
Q03B95_LACP3	LSEI_0687	0	0	0	2	3	3
Q03A10_LACP3	LSEI_1174	0	0	0	2	5	4
Q037U1_LACP3	LSEI_1759	0	0	0	3	2	2
ATPD_LACP3	LSEI_1163	0	0	0	1	3	2
Q03AX7_LACP3	LSEI_0809	0	0	0	2	3	4
Q039N6_LACP3	LSEI_1305	0	0	0	1	2	3
Q03BU1_LACP3	LSEI_0480	0	0	0	2	2	2
Q03B87_LACP3	LSEI_0695	0	0	0	2	1	2
Q03BD8_LACP3	LSEI_0643	0	0	0	1	2	2
Q03B32_LACP3	LSEI_0754	0	0	0	1	1	1
Q039P1_LACP3	LSEI_1298	0	0	0	1	1	1
RL13_LACP3	LSEI_2463	0	0	0	58	20	5
RS10_LACP3	LSEI_2504	0	0	0	14	21	55
RL17_LACP3	LSEI_2476	0	0	0	9	16	44
RS4_LACP3	LSEI_1244	0	0	0	12	38	21
RS6_LACP3	LSEI_0009	0	0	0	37	16	7
RS17_LACP3	LSEI_2494	0	0	0	3	25	34
S6C4R9_LACCA	LBCZ_0011	0	0	0	2	35	14

Q038N2_LACP3	LSEI_1566	0	0	0	7	9	23
EFTS_LACP3	LSEI_1585	0	0	0	4	12	15
Q03AF7_LACP3	LSEI_1019	0	0	0	4	14	10
Q034Q3_LACP3	LSEI_2598	0	0	0	18	3	2
Q039R4_LACP3	LSEI_1275	0	0	0	14	5	4
Q03CL8_LACP3	LSEI_0191	0	0	0	3	10	4
Q03C04_LACP3	LSEI_0412	0	0	0	2	5	9
RPOC_LACP3	LSEI_2515	0	0	0	6	3	10
Q035P9_LACP3	LSEI_2329	0	0	0	3	9	4
Q03BQ5_LACP3	LSEI_0517	0	0	0	0	17	1
METK_LACP3	LSEI_0877	0	0	0	10	1	6
Q033V0_LACP3	LSEI_2808	0	0	0	1	3	6
Q03AP5_LACP3	LSEI_0928	0	0	0	8	0	6
RL14_LACP3	LSEI_2493	0	0	0	1	6	9
EFG_LACP3	LSEI_2508	0	0	0	10	1	4
Q039Q5_LACP3	LSEI_1284	0	0	0	4	2	10
Q03AZ4_LACP3	LSEI_0792	0	0	0	5	5	1
Q03AC1_LACP3	LSEI_1057	0	0	0	2	8	3
Q034V9_LACP3	LSEI_2540	0	0	0	3	1	2
Q039D9_LACP3	LSEI_1405	0	0	0	3	1	2
Q03AI0_LACP3	LSEI_0996	0	0	0	0	3	6
Q039R0_LACP3	LSEI_1279	0	0	0	0	4	1
Q038C0_LACP3	LSEI_1679	0	0	0	0	5	1

Q034U8_LACP3	LSEI_2551	0	0	0	2	2	0
RL19_LACP3	LSEI_1597	0	0	0	0	1	0
Q03AS8_LACP3	LSEI_0894	0	0	0	3	1	4
Q036B2_LACP3	LSEI_2213	0	0	0	0	2	5
Q036H6_LACP3	LSEI_2147	0	0	0	6	0	1
RS12_LACP3	LSEI_2510	0	0	0	0	4	1
Y1299_LACP3	LSEI_1299	0	0	0	3	0	4
PURA_LACP3	LSEI_0122	0	0	0	0	0	1
RL6_LACP3	LSEI_2488	0	0	0	0	3	2
Q034P8_LACP3	LSEI_2603	0	0	0	1	1	4
Q034P5_LACP3	LSEI_2607	0	0	0	0	2	1
Q03B16_LACP3	LSEI_0770	0	0	0	3	1	1
Q039J6_LACP3	LSEI_1347	0	0	0	1	1	2
Q038U7_LACP3	LSEI_1499	0	0	0	2	0	2
RS3_LACP3	LSEI_2497	0	0	0	0	2	1
Q03D54_LACP3	LSEI_0002	0	0	0	1	2	0
Q035W6_LACP3	LSEI_2262	0	0	0	0	0	2
RS19_LACP3	LSEI_2499	0	0	0	0	0	4
Q03A05_LACP3	LSEI_1180	0	0	0	2	0	1
NAGB_LACP3	LSEI_2889	0	0	0	0	2	1
Q038E7_LACP3	LSEI_1652	0	0	0	1	0	1
COAA_LACP3	LSEI_1980	0	0	0	0	1	0
Q03BY3_LACP3	LSEI_0433	0	0	0	2	0	0

Q038X9_LACP3	LSEI_1465	0	0	0	0	2	0
MECA_LACP3	LSEI_1734	0	0	0	1	1	0
Q038M2_LACP3	LSEI_1576	0	0	0	0	1	1
GLMM_LACP3	LSEI_1018	0	0	0	1	0	0
