# Supplementary material for: Isolation and characterization of Lactobacillus-derived membrane vesicles
Source: Sci Rep. 2019 Jan 29;9:877. doi: 10.1038/s41598-018-37120-6 (PMC6351534; doi:10.1038/s41598-018-37120-6)
Supplement: Supplementary file 4 — tablse s3 [file 41598_2018_37120_MOESM4_ESM.pdf]

Table S3. List of proteins identified in MVs and pellet *Lactobacillus reuteri*.

Uniprot ID	Locus ID	MV sample 1	MV sample 2	MV sample 3	Pellet sample 1	Pellet sample 2	Pellet sample 3
A5VIM1_LACRD	Lreu_0426	223	137	318	25	41	52
A5VI92_LACRD	Lreu_0296	111	69	99	70	105	94
A5VML7_LACRD	Lreu_1853	94	31	121	8	4	9
OTC_LACRD	arcB	0	0	129	127	124	113
A5VJJ1_LACRD	Lreu_0751	43	0	76	119	120	154
A5VHI2_LACRD	Lreu_0030	34	0	83	132	102	123
NADK_LACRD	nadK	68	0	0	0	0	0
A5VIP4_LACRD	Lreu_0450	60	0	0	0	0	0
A5VKT5_LACRD	Lreu_1202	0	0	53	43	24	35
A5VM86_LACRD	cobD	34	0	0	0	0	0
A5VKG9_LACRD	Lreu_1086	17	15	0	0	0	0
A5VI82_LACRD	Lreu_0286	26	0	0	0	0	0
CH60_LACRD	groL	0	0	23	179	211	203
A5VKN8_LACRD	ffh	17	0	0	2	0	3
A5VLR9_LACRD	Lreu_1548	17	0	0	0	0	0
A5VKJ0_LACRD	Lreu_1107	17	0	0	0	0	0
A5VJF6_LACRD	ldh	0	0	15	76	47	77
EFTU_LACRD	tuf	0	0	0	1033	871	864
A5VM51_LACRD	Lreu_1686	0	0	0	446	411	507
A5VJK5_LACRD	Lreu_0765	0	0	0	492	437	336
A5VMD1_LACRD	Lreu_1766	0	0	0	358	298	403

A5VL58_LACRD	Lreu_1325	0	0	0	252	205	119
A5VHJ7_LACRD	Lreu_0045	0	0	0	157	174	176
A5VJK3_LACRD	Lreu_0763	0	0	0	135	210	107
A5VMA6_LACRD	Lreu_1741	0	0	0	136	133	133
A5VI89_LACRD	Lreu_0293	0	0	0	170	83	105
RL7_LACRD	rplL	0	0	0	119	173	55
A5VIB7_LACRD	Lreu_0321	0	0	0	101	92	127
RL17_LACRD	rplQ	0	0	0	141	113	56
EFG_LACRD	fusA	0	0	0	110	104	84
TIG_LACRD	tig	0	0	0	110	105	89
A5VMB2_LACRD	Lreu_1747	0	0	0	84	76	87
RL2_LACRD	rplB	0	0	0	81	130	49
A5VHS5_LACRD	pyrE	0	0	0	74	64	105
A5VMD0_LACRD	zwf	0	0	0	82	59	72
A5VMA0_LACRD	Lreu_1735	0	0	0	85	57	75
A5VMB4_LACRD	Lreu_1749	0	0	0	40	126	60
A5VLL8_LACRD	Lreu_1496	0	0	0	36	64	118
DNAK_LACRD	dnaK	0	0	0	57	70	72
A5VMA1_LACRD	Lreu_1736	0	0	0	52	35	93
A5VLS2_LACRD	Lreu_1553	0	0	0	69	44	69
Y1268_LACRD	Lreu_1268	0	0	0	48	76	65
PYRB_LACRD	pyrB	0	0	0	65	48	73
A5VIA5_LACRD	rplA	0	0	0	60	71	49

A5VM99_LACRD	Lreu_1734	0	0	0	70	33	77
RL5_LACRD	rplE	0	0	0	58	41	80
SYR_LACRD	argS	0	0	0	58	54	62
ATPB_LACRD	atpD	0	0	0	77	54	45
RL13_LACRD	rplM	0	0	0	41	45	91
ATPA_LACRD	atpA	0	0	0	44	47	61
A5VL40_LACRD	Lreu_1307	0	0	0	52	53	45
RS2_LACRD	rpsB	0	0	0	48	48	67
RL10_LACRD	rplJ	0	0	0	56	62	40
RBSD_LACRD	rbsD	0	0	0	65	58	21
RS7_LACRD	rpsG	0	0	0	32	65	41
A5VIT0_LACRD	Lreu_0486	0	0	0	59	41	35
A5VMN8_LACRD	Lreu_1875	0	0	0	33	85	35
A5VHQ0_LACRD	Lreu_0102	0	0	0	42	61	41
A5VM57_LACRD	Lreu_1692	0	0	0	37	34	36
RS4_LACRD	rpsD	0	0	0	31	34	47
A5VHY0_LACRD	Lreu_0184	0	0	0	37	27	51
EFTS_LACRD	tsf	0	0	0	23	51	44
A5VL73_LACRD	Lreu_1346	0	0	0	47	31	38
A5VIJ3_LACRD	Lreu_0398	0	0	0	37	42	36
RS3_LACRD	rpsC	0	0	0	36	31	46
A5VMB0_LACRD	Lreu_1745	0	0	0	37	50	40
A5VJ73_LACRD	Lreu_0632	0	0	0	28	34	40
RL11_LACRD	rplK	0	0	0	45	47	30
A5VL24_LACRD	Lreu_1291	0	0	0	40	36	32

A5VL46_LACRD	Lreu_1313	0	0	0	29	24	39
CH10_LACRD	groS	0	0	0	23	36	46
RPOC_LACRD	rpoC	0	0	0	38	17	33
RL18_LACRD	rplR	0	0	0	24	31	37
RPOA_LACRD	rpoA	0	0	0	27	31	42
A5VMB1_LACRD	Lreu_1746	0	0	0	32	36	28
ARCA_LACRD	arcA	0	0	0	36	30	31
A5VJ72_LACRD	Lreu_0631	0	0	0	43	15	30
RS8_LACRD	rpsH	0	0	0	33	22	20
A5VJ74_LACRD	Lreu_0633	0	0	0	25	25	30
A5VJ75_LACRD	Lreu_0634	0	0	0	31	26	28
RS5_LACRD	rpsE	0	0	0	24	27	30
A5VL18_LACRD	rbsK	0	0	0	23	20	30
EFP_LACRD	efp	0	0	0	27	35	22
A5VLZ7_LACRD	Lreu_1631	0	0	0	25	22	28
RL15_LACRD	rplO	0	0	0	20	34	23
RS19_LACRD	rpsS	0	0	0	30	42	13
RL3_LACRD	rplC	0	0	0	29	22	28
A5VI91_LACRD	Lreu_0295	0	0	0	30	19	31
RRF_LACRD	frr	0	0	0	18	30	22
A5VMM7_LACRD	thrS	0	0	0	22	27	23
A5VJM5_LACRD	Lreu_0785	0	0	0	20	31	21
A5VHS2_LACRD	pyrC	0	0	0	28	18	27
A5VIY3_LACRD	Lreu_0539	0	0	0	21	30	28

A5VMF7_LACRD	Lreu_1792	0	0	0	25	28	25
A5VHZ8_LACRD	Lreu_0202	0	0	0	16	34	26
A5VHU4_LACRD	gpmA	0	0	0	24	20	30
A5VJG9_LACRD	Lreu_0729	0	0	0	19	30	22
RL6_LACRD	rplF	0	0	0	21	13	24
RS13_LACRD	rpsM	0	0	0	18	21	19
G6PI_LACRD	pgi	0	0	0	21	28	10
A5VK26_LACRD	Lreu_0938	0	0	0	19	25	24
RL31B_LACRD	rpmE2	0	0	0	19	38	13
A5VIG7_LACRD	Lreu_0372	0	0	0	22	19	26
A5VMU1_LACRD	Lreu_1933	0	0	0	20	18	23
RPOB_LACRD	rpoB	0	0	0	23	13	19
A5VK77_LACRD	acpP	0	0	0	26	21	13
RL20_LACRD	rplT	0	0	0	18	18	24
RL23_LACRD	rplW	0	0	0	16	14	22
A5VMK5_LACRD	Lreu_1840	0	0	0	13	16	28
RL16_LACRD	rplP	0	0	0	18	22	20
RS10_LACRD	rpsJ	0	0	0	15	13	14
A5VMP6_LACRD	Lreu_1883	0	0	0	23	14	13
A5VLH2_LACRD	rpsI	0	0	0	25	19	14
UPP_LACRD	upp	0	0	0	17	19	22
RL22_LACRD	rplV	0	0	0	20	14	13
Y552_LACRD	Lreu_0552	0	0	0	11	15	17

A5VLH1_LACRD	Lreu_1449	0	0	0	18	18	23
A5VKF5_LACRD	Lreu_1072	0	0	0	15	18	21
RS6_LACRD	rpsF	0	0	0	14	11	19
A5VJI4_LACRD	Lreu_0744	0	0	0	18	13	20
A5VKU7_LACRD	greA	0	0	0	10	19	28
GUAA_LACRD	guaA	0	0	0	18	12	25
A5VJ04_LACRD	ackA	0	0	0	16	22	14
GPSB_LACRD	gpsB	0	0	0	10	27	15
ATPF_LACRD	atpF	0	0	0	12	18	25
A5VM23_LACRD	Lreu_1658	0	0	0	8	19	29
A5VMA9_LACRD	Lreu_1744	0	0	0	12	14	19
A5VIH5_LACRD	Lreu_0380	0	0	0	12	13	20
A5VJ78_LACRD	Lreu_0637	0	0	0	17	10	21
A5VKT9_LACRD	Lreu_1206	0	0	0	17	11	15
RS11_LACRD	rpsK	0	0	0	12	7	18
A5VL23_LACRD	Lreu_1290	0	0	0	13	18	15
A5VI17_LACRD	prs	0	0	0	16	15	20
A5VL60_LACRD	Lreu_1327	0	0	0	17	12	18
A5VIC3_LACRD	Lreu_0327	0	0	0	15	24	10
A5VID5_LACRD	Lreu_0339	0	0	0	8	7	14
DEOB_LACRD	deoB	0	0	0	14	16	13
RS12_LACRD	rpsL	0	0	0	13	15	18
A5VI67_LACRD	lysS	0	0	0	14	18	14

A5VLI4_LACRD	adk	0	0	0	16	18	12
A5VLN1_LACRD	Lreu_1510	0	0	0	7	19	15
A5VJ36_LACRD	ftsZ	0	0	0	12	12	16
RL27_LACRD	rpmA	0	0	0	11	10	22
A5VJ40_LACRD	Lreu_0597	0	0	0	10	15	9
RS20_LACRD	rpsT	0	0	0	11	15	19
A5VJD7_LACRD	nusA	0	0	0	16	12	12
A5VHR4_LACRD	Lreu_0116	0	0	0	13	15	14
A5VKR9_LACRD	Lreu_1186	0	0	0	11	12	19
A5VLJ6_LACRD	rpsQ	0	0	0	7	23	14
RL4_LACRD	rplD	0	0	0	11	11	15
RPOZ_LACRD	rpoZ	0	0	0	10	16	16
A5VIZ2_LACRD	Lreu_0548	0	0	0	13	18	10
SYE_LACRD	gltX	0	0	0	15	13	12
A5VHS4_LACRD	pyrF	0	0	0	17	15	8
A5VJH0_LACRD	Lreu_0730	0	0	0	7	13	20
A5VML5_LACRD	Lreu_1851	0	0	0	16	16	10
A5VLR7_LACRD	Lreu_1546	0	0	0	14	14	13
DEOD_LACRD	deoD	0	0	0	14	7	20
RS16_LACRD	rpsP	0	0	0	13	24	7
A5VI26_LACRD	pyrG	0	0	0	6	4	7
RL29_LACRD	rpmC	0	0	0	6	13	9
A5VM16_LACRD	Lreu_1650	0	0	0	19	7	10

A5VHQ9_LACRD	deoC	0	0	0	14	13	8
A5VIF9_LACRD	hpf	0	0	0	9	12	14
A5VK68_LACRD	Lreu_0981	0	0	0	10	6	17
A5VHX8_LACRD	Lreu_0182	0	0	0	10	11	17
SYL_LACRD	leuS	0	0	0	7	5	3
A5VKY4_LACRD	Lreu_1251	0	0	0	8	11	7
A5VM24_LACRD	Lreu_1659	0	0	0	10	6	13
SYN_LACRD	asnS	0	0	0	8	5	9
PYRDA_LACRD	pyrD	0	0	0	13	10	11
A5VMP4_LACRD	Lreu_1881	0	0	0	14	6	10
A5VJ10_LACRD	Lreu_0566	0	0	0	10	7	17
A5VMD4_LACRD	Lreu_1769	0	0	0	13	8	14
RL9_LACRD	rplI	0	0	0	11	12	13
A5VLS1_LACRD	Lreu_1552	0	0	0	6	10	13
A5VMB3_LACRD	Lreu_1748	0	0	0	8	16	9
A5VM98_LACRD	ackA	0	0	0	9	8	13
RL14_LACRD	rplN	0	0	0	7	3	8
A5VHK6_LACRD	Lreu_0054	0	0	0	9	7	10
A5VIX7_LACRD	alaS	0	0	0	11	4	10
RS21_LACRD	rpsU	0	0	0	8	10	15
A5VI65_LACRD	hslO	0	0	0	7	13	9
CLPP_LACRD	clpP	0	0	0	8	7	3
A5VI06_LACRD	metG	0	0	0	9	6	9

A5VJ21_LACRD	Lreu_0578	0	0	0	5	8	12
GLYA_LACRD	glyA	0	0	0	10	4	14
A5VIV3_LACRD	Lreu_0509	0	0	0	9	5	11
PYRH_LACRD	pyrH	0	0	0	7	6	10
IF1_LACRD	infA	0	0	0	6	15	6
A5VJ52_LACRD	prs	0	0	0	7	5	13
A5VI64_LACRD	ftsH	0	0	0	6	4	9
GATB_LACRD	gatB	0	0	0	10	4	9
IF2_LACRD	infB	0	0	0	9	3	6
A5VIR5_LACRD	Lreu_0471	0	0	0	8	2	9
A5VM54_LACRD	Lreu_1689	0	0	0	9	4	8
A5VLR5_LACRD	Lreu_1544	0	0	0	7	6	6
A5VHP9_LACRD	Lreu_0101	0	0	0	10	9	4
A5VKX4_LACRD	infC	0	0	0	6	8	9
A5VLS8_LACRD	Lreu_1559	0	0	0	9	2	6
A5VKP6_LACRD	Lreu_1163	0	0	0	8	4	8
A5VHQ7_LACRD	ychF	0	0	0	5	9	7
ATPD_LACRD	atpH	0	0	0	6	11	5
A5VKQ0_LACRD	Lreu_1167	0	0	0	6	9	7
Y534_LACRD	Lreu_0534	0	0	0	6	7	6
A5VIH1_LACRD	Lreu_0376	0	0	0	6	5	10
RPIA_LACRD	rpiA	0	0	0	7	7	4
A5VHP2_LACRD	Lreu_0091	0	0	0	6	6	9
A5VLL6_LACRD	Lreu_1494	0	0	0	6	2	8

A5VKW6_LACRD	rsfS	0	0	0	5	5	4
A5VIA3_LACRD	nusG	0	0	0	6	7	7
A5VM42_LACRD	Lreu_1677	0	0	0	7	6	3
A5VJ45_LACRD	Lreu_0602	0	0	0	6	4	9
A5VHX1_LACRD	Lreu_0175	0	0	0	5	5	7
A5VMA7_LACRD	Lreu_1742	0	0	0	4	5	4
SYP_LACRD	proS	0	0	0	6	4	7
GRPE_LACRD	grpE	0	0	0	4	6	6
A5VJY0_LACRD	Lreu_0891	0	0	0	6	4	6
A5VHF4_LACRD	Lreu_0002	0	0	0	6	2	7
A5VJI8_LACRD	pepT	0	0	0	4	1	3
A5VHL5_LACRD	Lreu_0064	0	0	0	6	2	4
A5VLQ9_LACRD	Lreu_1538	0	0	0	6	4	2
A5VHW2_LACRD	Lreu_0164	0	0	0	5	5	6
A5VIZ1_LACRD	Lreu_0547	0	0	0	5	6	5
A5VLQ5_LACRD	Lreu_1534	0	0	0	3	4	2
MURC_LACRD	murC	0	0	0	7	3	3
A5VL89_LACRD	Lreu_1364	0	0	0	5	4	5
A5VLG4_LACRD	gatC	0	0	0	5	5	4
A5VIX6_LACRD	cshB	0	0	0	6	4	3
DEF_LACRD	def	0	0	0	4	5	5
A5VIH6_LACRD	Lreu_0381	0	0	0	2	2	3
A5VKV0_LACRD	pheT	0	0	0	2	4	6

A5VLQ2_LACRD	fumC	0	0	0	6	2	4
A5VII9_LACRD	rnr	0	0	0	6	2	2
REX_LACRD	rex	0	0	0	5	2	4
DDL_LACRD	ddl	0	0	0	5	2	4
A5VK58_LACRD	Lreu_0970	0	0	0	4	3	3
A5VKZ8_LACRD	prsA	0	0	0	2	2	3
A5VHQ1_LACRD	Lreu_0103	0	0	0	4	4	2
SYC_LACRD	cysS	0	0	0	3	1	3
A5VMG7_LACRD	Lreu_1802	0	0	0	1	4	4
A5VIJ9_LACRD	rbsK	0	0	0	25	21	63
A5VIV6_LACRD	Lreu_0512	0	0	0	35	2	23
A5VMM4_LACRD	Lreu_1860	0	0	0	10	13	35
A5VMP5_LACRD	Lreu_1882	0	0	0	10	7	28
RL19_LACRD	rplS	0	0	0	8	5	29
PDUL_LACRD	pduL	0	0	0	6	10	25
A5VJ90_LACRD	rnj	0	0	0	1	0	0
A5VIP6_LACRD	prfA	0	0	0	4	8	0
A5VK46_LACRD	Lreu_0958	0	0	0	13	1	11
SYGB_LACRD	glyS	0	0	0	9	3	16
A5VKP3_LACRD	acpP	0	0	0	6	19	3
A5VIU8_LACRD	valS	0	0	0	9	2	15
SYY_LACRD	tyrS	0	0	0	6	3	16
RS15_LACRD	rpsO	0	0	0	1	2	8

RL28_LACRD	rpmB	0	0	0	3	0	12
A5VI18_LACRD	Lreu_0222	0	0	0	5	7	17
RECA_LACRD	recA	0	0	0	6	0	10
A5VI90_LACRD	Lreu_0294	0	0	0	0	10	6
A5VMA2_LACRD	Lreu_1737	0	0	0	5	4	13
GATA_LACRD	gatA	0	0	0	11	2	7
RL24_LACRD	rplX	0	0	0	6	0	0
ATPG_LACRD	atpG	0	0	0	5	1	12
SYD_LACRD	aspS	0	0	0	7	0	8
RS18_LACRD	rpsR	0	0	0	8	5	0
A5VMC7_LACRD	Lreu_1762	0	0	0	6	4	0
RL21_LACRD	rplU	0	0	0	6	0	7
A5VML1_LACRD	Lreu_1847	0	0	0	6	0	8
A5VJY4_LACRD	Lreu_0895	0	0	0	9	2	2
A5VHY2_LACRD	Lreu_0186	0	0	0	6	0	7
FTHS_LACRD	fhs	0	0	0	8	0	4
RL30_LACRD	rpmD	0	0	0	0	0	9
A5VJI3_LACRD	sigA	0	0	0	4	8	2
A5VKY5_LACRD	Lreu_1252	0	0	0	0	7	6
A5VJ50_LACRD	Lreu_0607	0	0	0	2	12	0
A5VM43_LACRD	Lreu_1678	0	0	0	4	0	6
A5VLB6_LACRD	Lreu_1391	0	0	0	2	0	5
A5VIZ4_LACRD	Lreu_0550	0	0	0	4	0	3

GSA_LACRD	hemL	0	0	0	5	0	6
A5VI00_LACRD	trpS	0	0	0	2	0	8
A5VHP1_LACRD	Lreu_0090	0	0	0	5	0	7
A5VIS2_LACRD	Lreu_0478	0	0	0	3	0	6
SYS_LACRD	serS	0	0	0	4	2	0
A5VK75_LACRD	Lreu_0988	0	0	0	3	3	0
A5VJ41_LACRD	ileS	0	0	0	5	0	4
A5VL97_LACRD	Lreu_1372	0	0	0	0	3	0
A5VJY1_LACRD	Lreu_0892	0	0	0	5	0	0
A5VJC0_LACRD	Lreu_0679	0	0	0	0	5	4
A5VM59_LACRD	Lreu_1694	0	0	0	0	7	0
A5VMD3_LACRD	Lreu_1768	0	0	0	0	0	3
A5VIK1_LACRD	Lreu_0406	0	0	0	6	0	2
A5VHG7_LACRD	Lreu_0015	0	0	0	0	1	6
GPDA_LACRD	gpsA	0	0	0	3	0	4
A5VI40_LACRD	cshA	0	0	0	4	0	7
A5VI48_LACRD	dltA	0	0	0	4	0	2
GLMM_LACRD	glmM	0	0	0	1	0	8
A5VK73_LACRD	Lreu_0986	0	0	0	0	7	0
A5VJZ9_LACRD	Lreu_0911	0	0	0	2	0	0
SECA_LACRD	secA	0	0	0	2	0	2
A5VHG0_LACRD	Lreu_0008	0	0	0	2	3	0
A5VK79_LACRD	Lreu_0992	0	0	0	5	1	0

NADE_LACRD	nadE	0	0	0	4	0	0
RBFA_LACRD	rbfA	0	0	0	0	5	0
A5VLG8_LACRD	Lreu_1446	0	0	0	2	0	6
SYFA_LACRD	pheS	0	0	0	0	4	0
A5VKF9_LACRD	Lreu_1076	0	0	0	2	2	0
URK_LACRD	udk	0	0	0	3	0	3
A5VLM6_LACRD	Lreu_1505	0	0	0	2	0	0
A5VMG2_LACRD	Lreu_1797	0	0	0	3	0	2
A5VHW0_LACRD	Lreu_0162	0	0	0	2	0	2
A5VIU6_LACRD	Lreu_0502	0	0	0	3	0	0
A5VLD3_LACRD	Lreu_1408	0	0	0	2	0	0
A5VJH8_LACRD	era	0	0	0	2	0	2
A5VHF5_LACRD	Lreu_0003	0	0	0	1	0	0
A5VKT7_LACRD	Lreu_1204	0	0	0	0	2	0
A5VI63_LACRD	Lreu_0267	0	0	0	1	0	3
A5VMC1_LACRD	Lreu_1756	0	0	0	1	0	3
A5VIA6_LACRD	Lreu_0310	0	0	0	3	0	3
A5VL92_LACRD	Lreu_1367	0	0	0	0	0	3
A5VLQ1_LACRD	Lreu_1530	0	0	0	2	0	3
A5VKQ9_LACRD	gmk	0	0	0	2	0	4
A5VKU6_LACRD	Lreu_1213	0	0	0	2	4	0
A5VHQ3_LACRD	Lreu_0105	0	0	0	1	0	2
A5VJ35_LACRD	ftsA	0	0	0	3	0	0

A5VM66_LACRD	Lreu_1701	0	0	0	3	0	1
A5VHK7_LACRD	Lreu_0055	0	0	0	0	0	1
A5VM97_LACRD	Lreu_1732	0	0	0	0	3	0
A5VLS9_LACRD	Lreu_1560	0	0	0	3	0	0
A5VLR0_LACRD	Lreu_1539	0	0	0	2	2	0
A5VJI0_LACRD	glyQ	0	0	0	2	0	0
A5VI46_LACRD	dltC	0	0	0	0	2	0
A5VL99_LACRD	Lreu_1374	0	0	0	3	0	0
A5VIT5_LACRD	Lreu_0491	0	0	0	0	2	0
XPT_LACRD	xpt	0	0	0	0	1	1
A5VKE2_LACRD	Lreu_1058	0	0	0	1	4	0
A5VK72_LACRD	fabZ	0	0	0	0	0	3
A5VI93_LACRD	Lreu_0297	0	0	0	2	0	0
RF3_LACRD	prfC	0	0	0	0	1	0
A5VKW1_LACRD	Lreu_1228	0	0	0	0	0	2
A5VJK6_LACRD	Lreu_0766	0	0	0	0	0	2
A5VMK2_LACRD	Lreu_1837	0	0	0	0	0	2
A5VJD8_LACRD	Lreu_0697	0	0	0	2	0	0
A5VKT3_LACRD	Lreu_1200	0	0	0	3	0	2
A5VIB9_LACRD	Lreu_0323	0	0	0	2	1	0
RISB_LACRD	ribH	0	0	0	0	0	2
A5VIK5_LACRD	glmS	0	0	0	1	0	0
A5VHR5_LACRD	Lreu_0117	0	0	0	0	0	2

DAPA_LACRD	dapA	0	0	0	0	2	0
A5VK71_LACRD	Lreu_0984	0	0	0	3	0	0
MNMA_LACRD	mnmA	0	0	0	1	0	1
A5VIZ3_LACRD	Lreu_0549	0	0	0	0	2	0
A5VK76_LACRD	Lreu_0989	0	0	0	0	0	2
A5VIR9_LACRD	Lreu_0475	0	0	0	0	1	0
MURG_LACRD	murG	0	0	0	0	1	0
A5VHF8_LACRD	gyrA	0	0	0	2	0	0
METK_LACRD	metK	0	0	0	1	0	2
OBG_LACRD	obg	0	0	0	0	0	2
A5VL06_LACRD	Lreu_1273	0	0	0	0	0	3
A5VL78_LACRD	Lreu_1352	0	0	0	1	0	0
A5VME5_LACRD	Lreu_1780	0	0	0	0	0	3
A5VL04_LACRD	Lreu_1271	0	0	0	0	0	2
A5VI39_LACRD	murF	0	0	0	1	0	0
A5VI32_LACRD	Lreu_0236	0	0	0	1	0	0
A5VHQ5_LACRD	Lreu_0107	0	0	0	2	0	0
A5VLN7_LACRD	rnj	0	0	0	1	0	0
A5VI84_LACRD	Lreu_0288	0	0	0	1	0	0
A5VI85_LACRD	Lreu_0289	0	0	0	1	0	0
A5VIU5_LACRD	ezrA	0	0	0	1	0	0
A5VHJ4_LACRD	Lreu_0042	0	0	0	1	0	0
A5VI88_LACRD	Lreu_0292	0	0	0	0	0	1

A5VHX0_LACRD	Lreu_0174	0	0	0	1	0	0
A5VK66_LACRD	Lreu_0979	0	0	0	0	1	0
GAL1_LACRD	galK	0	0	0	1	0	0
A5VKY8_LACRD	Lreu_1255	0	0	0	1	0	0
LUXS_LACRD	luxS	0	0	0	1	0	0
A5VM60_LACRD	Lreu_1695	0	0	0	1	0	0
A5VME1_LACRD	galT	0	0	0	1	0	0
A5VI79_LACRD	Lreu_0283	0	0	0	0	0	1
